# Active vitamin D increases myogenic differentiation in C2C12 cells via a vitamin D response element on the myogenin promoter

**DOI:** 10.3389/fphys.2023.1322677

**Published:** 2024-01-08

**Authors:** Kathryn H. Alliband, Tim Parr, Preeti H. Jethwa, John M. Brameld

**Affiliations:** Division of Food, Nutrition and Dietetics, School of Biosciences, University of Nottingham, Sutton Bonington Campus, Loughborough, United Kingdom

**Keywords:** vitamin D, vitamin D response element, vitamin D receptor, myogenin, differentiation, myogenesis

## Abstract

**Background:** Skeletal muscle development during embryogenesis depends on proliferation of myoblasts followed by differentiation into myotubes/multinucleated myofibers. Vitamin D (VD) has been shown to affect these processes, but there is conflicting evidence within the current literature on the exact nature of these effects due to a lack of time course data. With 20%–40% of pregnant women worldwide being VD deficient, it is crucial that a clearer understanding of the impact of VD on myogenesis is gained.

**Methods:** A detailed 8-day differentiation time course was used where C2C12 cells were differentiated in control media (2% horse serum) or with different concentrations of active VD, 1,25 (OH)_2_D_3_ (10^−13^ M, 10^−11^ M, 10^−9^ M or 10^−7^ M), and measurements were taken at 6 time points. DNA, creatine kinase and protein assays were carried out as well as quantitative PCR to determine expression of Myf5, MyoD, myogenin, MHC I, and MHC neonatal, MHC embryonic, MHC IIa, MHC IIx, and MHC IIb mRNAs. Transfections were carried out using one vector containing the myogenin promoter and another containing the same promoter with a 3 base mutation within a putative vitamin D response element (VDRE) to determine effects of 1,25 (OH)_2_D_3_ on myogenin transcription. Finally, a ChIP assay was performed to determine whether the VD receptor (VDR) binds to the putative VDRE.

**Results:** 1,25(OH)_2_D_3_ caused an inhibition of proliferation and an increase in differentiation in C2C12 cells. Myf5, myogenin, MHC I, and MHC neonatal, MHC embryonic, MHC IIa, MHC IIx, and MHC IIb expression were all increased by 1,25(OH)_2_D_3_. Myotube size was also increased by VD. When the putative VDRE on the myogenin promoter was mutated, the increase in expression by VD was lost. ChIP analysis revealed that the VDR does bind to the putative VDRE on the myogenin promoter.

**Conclusion:** Active VD directly increases myogenin transcription via a functional VDRE on the myogenin promoter, resulting in increased myogenic differentiation, increased expression of both the early and late MHC isoforms, and also increased myotube size. These results highlight the importance of VD status during pregnancy for normal myogenesis to occur, but further *in vivo* work is needed.

## 1 Introduction

It is well documented that vitamin D (VD) deficiency, defined in the UK by serum levels of inactive VD [25(OH)D_3_] falling below 25 nmol/L (Griffin et al., 2021), is associated with muscle weakness, and that this is reversible with dietary supplementation (Braga et al., 2017), indicating a link between serum 25(OH)D_3_ status and muscle function. There is also evidence of vitamin D receptor (VDR) expression in human muscle cells, as well as effects of VD treatment, suggesting that the effects on muscle may be direct and mediated through the VDR (Olsson et al., 2016). Furthermore, VDR knockout mice have smaller muscle fibers than wild type mice at 3 weeks of age, which becomes even more prominent by 8 weeks (Endo et al., 2003). Collectively, these findings suggest that VD and the VDR play an important role in the regulation of skeletal muscle function and development.

The biologically active form of VD, 1,25(OH)_2_D_3_ (also called calcitriol), has the highest affinity for the VDR with a k_D_ of 0.1 nM (Carlberg, 2019). The VDR belongs to the zinc finger class of transcription factors (Molnár, 2014), which can be activated by low (nanomolar) concentrations of 1,25(OH)_2_D_3_, and interacts in a sequence specific manner with genomic DNA (Carlberg and Campbell, 2013). Upon binding of 1,25(OH)_2_D_3_ to the VDR, a conformational change occurs allowing the VDR to bind to other proteins to form a dimer (Thompson et al., 1998). Hence, the VDR either forms a homodimer with another VDR, or a heterodimer by binding with a retinoic acid receptor (RAR) or a retinoid X receptor (RXR), with the latter being the predominant heterodimer formed (Carlberg and Molnár, 2015). Once formed, the homo- or heterodimer binds to a VD response element (VDRE) on a target gene, which are typically comprised of two conserved hexameric repeats separated by a 3-nucleotide spacer (Tsoumpra et al., 2020). In order for successful activation of transcription, the activated VDR needs to cause efficient dissociation of co-repressors as well as efficient association of co-activators (Carlberg and Molnár, 2015). Primarily, p160 coactivators and steroid receptor activators 1, 2, and 3 are recruited, which have histone acetylase activity resulting in the unwinding of DNA from the histones and allowing access for the transcriptional machinery (Christakos et al., 2016). In total, over 900 genes have been found to be regulated by VD (Wang et al., 2005).

Within the genes that have been shown to be regulated by VD, some genes relating to myogenesis have been identified (Girgis et al., 2014). Our recent systematic review (Alliband et al., 2021), evaluating the effects of active VD on muscle differentiation and expression of myogenic genes, showed a consistent anti-proliferative effect of active VD on muscle cells in culture, as well as an increase in myotube size. However, the effects on differentiation and the mRNA expression of myogenic regulatory factors (MRFs) and myosin heavy chain (MHC) isoforms were inconsistent. The main issue across the studies appeared to be a lack of time course data, with most only measuring gene expression at one or two time points, with large inconsistencies as to which day of differentiation this was done on. There was also variability in the concentrations of active VD used.

**FIGURE 1:**
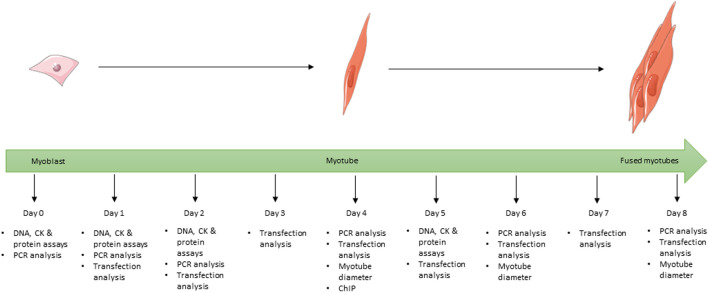
Schematic showing the full 8-day time course used for the differentiation of C2C12 cells during the study and at which time point each experiment was undertaken for all cells grown in differentiation media. Images used within this figure were obtained from smart servier medical art and can be found at https://smart.servier.com.

Hence, the aim of this study was to determine the effects of 1,25(OH)_2_D_3_ on muscle cell proliferation and differentiation, using the C2C12 muscle cell line, with the effects on differentiation being via a detailed time course for mRNA expression of the MRFs and MHC isoforms (Figure 1). This builds on our previous work which mapped the time course of mRNA expression of these genes during normal C2C12 differentiation (Brown et al., 2012). We used four different concentrations of 1,25(OH)_2_D_3_ which ranged from below physiological to above physiological concentrations (physiological range for 1,25(OH)_2_D_3_ = 4.3 × 10^−11^ M to 1.9 × 10^−10^ M) (Pagana et al., 2019) to determine whether any observed effects were dose dependent, and further investigated the mechanism for the observed effects of 1,25(OH)_2_D_3_ on myogenic differentiation.

## 2 Materials and methods

### 2.1 Cell culture

The C2C12 mouse myoblast cell line was obtained from the European Collection of Authenticated Cell Cultures (ECACC). C2C12 myoblasts were grown in growth medium consisting of Dulbecco’s modified eagle’s medium (DMEM; Sigma Aldrich, Poole, UK) supplemented with 10% (v/v) fetal bovine serum (FBS; Invitrogen, Paisley, UK) and 1% (v/v) 100 × penicillin streptomycin (P/S; Invitrogen, Paisley, UK) and incubated at 37°C and 5% CO_2_. Cells were treated with different concentrations of 1,25(OH)_2_D_3_ [either 10^−13^ M, 10^−11^ M, 10^−9^ M or 10^−7^ M in 0.1% ethanol (v/v)]. Two controls were used, one treated with 0.1% (v/v) ethanol and the second with normal differentiation media to determine whether the vehicle was causing any of the effects.

For the proliferation experiment, cells were plated onto 96-well plates (at 5,000 cells/well) prior to 1,25(OH)_2_D_3_ treatments being added 24 h later. Plates were stopped at t = 0 (24 h after plating, before addition of treatments) and at 24 h and 48 h post 1,25(OH)_2_D_3_ treatment by removing media and washing with phosphate buffered saline (PBS). 100 µL of dH_2_O was then added to each well and plates were frozen at −20°C prior to freeze-thawing and DNA assays.

For differentiation studies, cells were plated onto 6-well plates (1.5 × 10^5^ cells/well). On day 0 (approximately 70% confluence) cells underwent serum starvation to encourage differentiation by switching to differentiation media consisting of DMEM supplemented with 2% (v/v) horse serum (HS; Invitrogen, Paisley, UK) and 1% (v/v) 100 X P/S. VD treatments were also added at this point. Cells were cultured for up to 8 days and media was replaced every 48 h. Cells for protein and creatine kinase assays were harvested on days 0, 1, 2, and 5 (Figure 1) by scraping into 500 µL of tri-sodium citrate (0.05M, pH 6.8, 4°C) and placed on ice, then stored at −20°C. Cells for gene expression analysis were harvested on days 0, 1, 2, 4, 6, and 8 (Figure 1) by scraping into 200 µL of ice cold RNase free PBS and stored at −80°C prior to RNA extraction (see below).

### 2.2 DNA, protein and creatine kinase assays

To lyse the cells prior to DNA assays, plates underwent two freeze/thaw cycles. DNA contents were determined using the Hoechst fluorescent dye method as previously described (Rago et al., 1990; Hurley et al., 2006). In brief, 100 µL of working dye solution was added to 100 μL cell lysates on the 96-well plates. A standard curve of known concentrations of calf thymus DNA was run alongside the samples. Fluorescence was measured using a fluorescent plate reader (Fluostar Optima, BMG Labtech) at excitation of 350 nm and emission 460 nm.

For protein and creatine kinase assays, cells in tri-sodium citrate buffer were thawed on ice and then sonicated (Soniprep150, MSE, UK) using 10 µm amplitude for roughly 30 s, or until the sample was clear. Cell lysates were used immediately after sonication for the creatine kinase assay and were stored at −20°C again prior to protein and DNA assays. Creatine kinase activity (U/l) was determined as previously described (Hurley et al., 2006; Brown et al., 2012) using a CK kinetic assay kit (Thermo Electron Corporation, Fisher Scientific, Loughborough, UK). Samples were run on a 96-well plate and absorbance at 340 nm was read every 30 s for a total of 5 min at 37°C to determine enzyme activity (BioRad 680XR Microplate reader). Protein contents were determined using the Lowry method as previously described (Hurley et al., 2006; Brown et al., 2012). Samples were run on a 96-well plate alongside a standard curve of known concentrations of bovine serum albumin and absorbance was measured at 655 nm (BioRad 680XR Microplate reader).

A DNA assay was also carried out (as above) on these samples to normalise protein and CK activity against.

### 2.3 Gene expression analysis via quantitative RT-qPCR

An RNA isolation kit (ROCHE high pure isolation kit) was used according to the manufacturer’s protocol to extract total RNA from cells previously scraped into 200 µL RNase free PBS, after thawing on ice. Total RNA concentrations were determined using a Nanodrop ND-1000 (Thermo Scientific, Wilmington, United States). All samples were diluted to 50 ng/μL and stored at −80°C. First strand cDNA synthesis was carried out using a Thermo Scientific RevertAid RT kit (Thermo Scientific, Vilnius, Lithuania) according to manufacturer’s protocol using 500 ng total RNA per sample. First strand cDNA samples were then diluted 5-fold using RNase free water and stored at −20°C. A LightCycler^®^ 480 (Roche, Burgess Hill, UK) was used to carry out RT-qPCR analysis. Each well on a 384 well PCR plate contained the following: 5 µL first strand cDNA, 7.5 µL SYBR Green I Master mix (Roche, Burgess Hill, UK), 0.45 µL forward primer (10 µM), 0.45 µL reverse primer (10 µM), 1.6 µL RNase free H_2_O. The PCR amplification protocol was as follows: pre-incubation at 95°C for 5 min followed by 45 amplification cycles (denaturation of strands at 95°C for 10 s, annealing at 60°C for 15 s, elongation at 72°C for 15 s) (Brown et al., 2012). All reactions were carried out in triplicate. Initially, qPCR analysis was run on every cDNA sample using primers for a potential reference gene (TATA binding protein) to check that the crossing point (CP) for each sample was within 2 standard deviations of the mean of all samples. Once this was confirmed, a cDNA pool was made by combining 10 µL aliquots from every sample. This pool was then used in serial dilutions to create a standard curve to determine the PCR efficiencies for all primer pairs/genes and subsequently the transcript concentrations of the samples. Melt curve analysis was used to ensure only one peak was present (indicating a single amplicon product) for all primers in all samples. Supplementary Data S1 includes a list of genes measured and their corresponding forward and reverse primers. All PCR primers were designed to cross exon-exon boundaries and working primers were diluted from stock primers.

### 2.4 Oligreen quantification of total cDNA

As in our previous study (Brown et al., 2012), we were unable to find a reference gene that did not significantly change across the time course studied (data not shown). Therefore, an Oligreen ssDNA Assay kit (Invitrogen, Eugene, Oregon) was used to quantify cDNA content for each sample on a 384 well PCR plate according to manufacturer’s protocol. Fluorescence was quantified using the LightCycler^®^ 480 (Roche, Burgess Hill, UK) with increasing temperature at a continuous ramp rate of 0.11°C/s until it reached 95°C. Fluorescence measurements at 80°C were plotted against a cDNA standard curve (as described above) to determine relative cDNA quantity in each sample and this was subsequently used for normalisation of all genes.

### 2.5 Generation of myogenin promoter plasmids

The mouse myogenin promoter sequence was obtained from the NCBI database (accession M95800). The typical VDRE sequence obtained from (Tsoumpra et al., 2020) was aligned to the myogenin promoter sequence using Clustal Omega, which identified a potential VDRE (12/15 bases, 80% match) on the myogenin promoter at −1,260 to −1,246 from the transcription start site (TSS). A vector containing the mouse myogenin promoter sequence, including the potential VDRE, linked to a Turbo-green fluorescent protein (GFP) reporter (MyoG-VDRE-GFP) was purchased from VectorBuilder. Another vector with a mutated VDRE (MyoG-mutVDRE-GFP) was also purchased (VectorBuilder) which was identical in sequence apart from 3 bases in positions 2, 3, and 4 of the VDRE sequence (−1,261 to −1,264) which were changed from GAA to AGG. Full vector components and sequences are shown in Supplementary Data S2. Plasmids were prepared using a Promega PureYield Plasmid Miniprep kit (Madison, Wisconsin, USA) according to manufacturer’s protocol. Plasmid concentrations were measured using a nanodrop^®^ ND-1000 (Thermo Scientific, Wilmington, United States) and were stored at −20°C.

### 2.6 Myogenin promoter transfection studies

Transfection of C2C12 cells with the different vectors was carried out at 60%–70% confluency on day −1 (i.e. 24 h before switching to differentiation media). C2C12 cells were cultured in 24-well plates, at 30,000 cells/well, and were transfected 24 h after plating (whilst in the log phase of growth) using Lipofectamine 2000 (Invitrogen, California, United States) according to manufacturer’s protocol. Each well contained 500 ng vector (400 ng MyoG-GFP vector plus 100 ng pDsRed-Express-NI vector with a CMV promoter followed by red fluorescence protein (RFP) (Brown et al., 2014). This was to check for transfection efficiency after 24 h due to myogenin not being expressed until mid-stage differentiation and so the MyoG-GFP cells did not appear green until day 2. 2.5 µL lipofectamine in Opti-MEM medium (Gibco, Paisley, UK) was used per well. 30 min prior to cell imaging, media was replaced with Fluorobrite DMEM (Fisher Scientific, Loughborough, UK) with 2 drops per ml of NucBlue Hoechst nuclear stain (Fisher Scientific, Loughborough, UK) and incubated at 37°C, 5% CO_2_. Cell images were taken on a fluorescent microscope (Evos FL) and fluorescence was measured every 24 h for 8 days with a fluorescent plate reader (Fluostar Optima, BMG Labtech) using the matrix scan setting. This is where 30 × 30 measurements are taken over a 1.5 cm^2^ area for each well, and the average for each well calculated. GFP was measured at an excitation of 485 nm and emission of 520 nm and NucBlue was measured at an excitation of 355 nm and emission of 460 nm. All GFP data was normalised to NucBlue to account for any differences in cell number.

Images of GFP transfected myotubes were analysed for myotube diameter using ImageJ as described previously (Brown et al., 2018; Brearley et al., 2021). Briefly, four images at ×10 magnification were used for each treatment at each time point and five myotubes were analysed per image, a total of 20 myotubes per treatment at each time point. Myotube diameter was measured at 25%, 50%, and 75% of total myotube length to provide an average diameter per myotube. Length in number of pixels was converted to µM by measuring the length of a 400 µM scale bar in pixels.

### 2.7 Chromatin immunoprecipitation (ChIP) assay

Cells were plated on 6 well plates at 1.5 × 10^5^ cells/well and cultured in growth medium for 48 h until 70% confluent. Media was then removed and replaced with differentiation media supplemented with either 0.1% (v/v) EtOH, 10^−11^ M or 10^−7^ M 1,25(OH)_2_D_3_ for 4 days, with media changed every 48 h 4 days after switching to differentiation media, cells were cross linked using 1% (v/) formaldehyde. ChIP assay was carried out using a Pierce Agarose ChIP kit (Pierce, Thermo Scientific, Wilmington, United States). According to manufacturer’s protocol. After digestion, genomic DNA fragment size was checked by running on a 1% (w/v) agarose gel to ensure fragments were between 200–1000 bp. Anti-vitamin D receptor antibody (ab3508 Abcam, UK) was used to isolate genomic DNA fragments containing a VDRE. The myogenin promoter VDRE primers (Supplementary Data S1) used for PCR were designed using Primer Express. Each PCR reaction contained the following: 5 µL genomic DNA, 7.5 µL SYBR Green I Master mix (Roche, Burgess Hill, UK), 0.45 µL forward primer (10 µM), 0.45 µL reverse primer (10 µM), 1.6 µL RNase free H_2_O and PCR conditions were the same as described above. PCR products were run on a 2.5% (w/v) Metaphor Agarose gel in 1 × TAE buffer at 100 V to confirm fragment size.

### 2.8 Statistical analysis

Four biological replicates (i.e., wells) were used throughout. Assays and PCR analyses were carried out in triplicate for each sample. Statistical analyses were carried out using GenStat 22nd edition. Most data were analysed by 2-way ANOVA (day x treatment) with the exception of the second promoter transfection experiment where two different vectors were used so a 3-way ANOVA (day x treatment x vector) was carried out. *Post hoc* Bonferroni tests were then used to identify groups that were significantly different. Results were deemed significant if *p* < 0.05.

## 3 Results

### 3.1 Vitamin D decreases proliferation and increases differentiation of C2C12 cells

**FIGURE 2:**
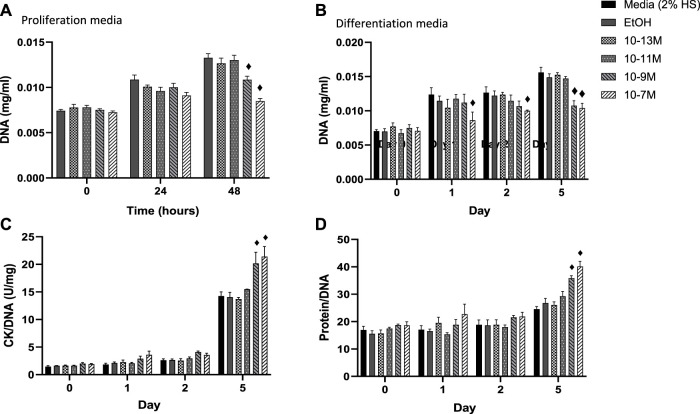
**(A)** Effects of 1,25 (OH)_2_D_3_ treatment in C2C12 cells on DNA, creatine kinase and protein. **(A)** DNA (mg/mL) of C2C12 cells grown in proliferation media for 48 h with varying concentrations of 1,25 (OH)_2_D_3_ in 0.1% (v/v) ethanol. **(B–D)** C2C12 cells grown in differentiation media for 5 days with varying concentrations of 1,25(OH)_2_D_3_ in 0.1% (v/v) ethanol. **(B)** DNA (mg/mL) **(C)** Creatine Kinase activity normalised to DNA (U/L) **(D)** Protein content normalised to DNA. ♦ indicates where a bar is significantly different (*p* < 0.05) to both media and ethanol controls [media (2% HS) and EtOH] within the same time point. Error bars show standard deviation. 4 biological replicates (wells) were used throughout and all assays were carried out in triplicate. Statistical analysis was carried out via a 2-way ANOVA with a *post hoc* Bonferroni test where time*treatment interaction was significant (*p* < 0.05).

In order for myoblasts to undergo myogenic differentiation they must first exit from the cell cycle so that they can switch from a proliferative to a differentiative state (Kitzmann and Fernandez, 2001). DNA content of C2C12 cells was measured as a marker of cell proliferation with a decrease in DNA content indicating a decrease in cell number. A significant day × treatment interaction was observed for DNA content (*p* = 0.001) for cells in proliferation media, Figure 2A. The two highest concentrations of 1,25(OH)_2_D_3_ resulted in significantly lower levels of DNA at 48 h compared to controls (*p* < 0.05) but were similar to DNA levels at the time 0 point. For cells in differentiation media a significant day × treatment interaction was also observed for DNA content (*p* < 0.001), Figure 2B. By day 1 (24 h post switching to differentiation media) cells treated with 10^−7^ M 1,25(OH)_2_D_3_ had significantly lower DNA content than controls (*p* < 0.05) indicating decreased proliferation. By day 5, both 10^−7^ M and 10^−9^ M 1,25(OH)_2_D_3_ had significantly lower DNA content than day 5 controls (*p* < 0.05).

Within differentiating muscle cells in culture, creatine kinase (CK) begins to be expressed after proliferating myoblasts exit from the cell cycle and begin to express differentiation specific factors (Tai et al., 2011). Therefore, CK enzyme activity was measured as a marker of mid stage differentiation (Figure 2C). There was a significant day × treatment interaction (*p* < 0.001) for CK activity. Activity did not increase between day 0 and 2 for all treatment groups, but CK enzyme activity for both 10^−9^ M and 10^−7^ M 1,25(OH)_2_D_3_ was significantly higher than controls by day 5 (*p* < 0.05).

A significant day × treatment interaction was also seen for the protein/DNA ratio in response to 1,25(OH)_2_D_3_ treatment (*p* < 0.01, Figure 2D). Again, there were no differences until day 5, when both 10^−7^ M and 10^−9^ M 1.25(OH)_2_D_3_ resulted in significantly higher protein contents than controls (*p* < 0.05). The increases in both CK enzyme activity and protein contents, relative to DNA, indicate a significant increase in differentiation with both 10^−7^ M and 10^−9^ M 1,25(OH)_2_D_3_ compared to the controls.

### 3.2 Vitamin D influences mRNA expression of two myogenic regulatory factors

**FIGURE 3:**
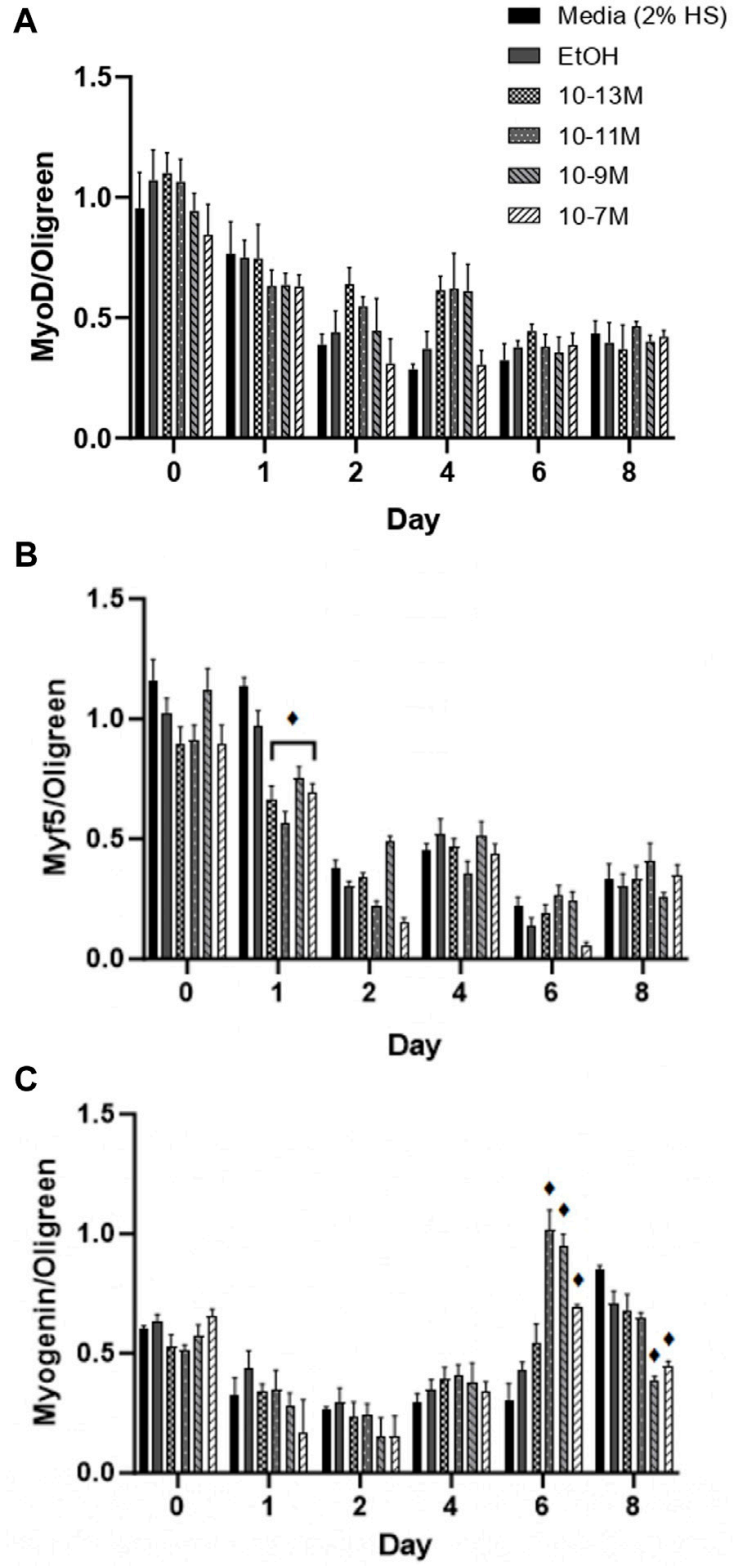
Effects of 1,25 (OH)_2_D_3_ on C2C12 cells over 8 days on myogenic regulatory factor gene expression. **(A)** MyoD **(B)** Myf5 and **(C)** Myogenin mRNA expression normalised to Oligreen during C2C12 differentiation over an 8-day time course with varying concentrations of 1,25(OH)_2_D_3_ dissolved in 0.1% (v/v) ethanol. ♦ symbol indicates where a bar is significantly different (*p* < 0.05) to both media and ethanol controls [media (2% HS) and EtOH] within the same time point. Error bars show standard deviation. 4 biological replicates (wells) were used throughout and all PCR experiments were carried out in triplicate. Statistical analysis was carried out via a 2-way ANOVA with a *post hoc* Bonferroni test where time*treatment interaction was significant (*p* < 0.05).

Myf5 and MyoD are myogenic regulatory factors (MRFs) responsible for cell commitment to the myogenic lineage, exit from the cell cycle and the initiation of terminal differentiation (Montenegro et al., 2019) and therefore are markers of early stage differentiation. For MyoD mRNA expression (Figure 3A), there was no significant day × treatment interaction (*p* = 0.49) and no significant effect of treatment (*p* = 0.12). As expected, there was a significant effect of time (*p* < 0.001) with mRNA expression decreasing from day 0 to day 1 (*p* < 0.05) and from day 1 to day 2 (*p* < 0.05). For Myf5 mRNA expression (Figure 3B) there was a significant day × treatment interaction (*p* < 0.001). As expected, Myf5 mRNA expression decreased over time for all treatment groups, but all 1,25(OH)_2_D_3_ treatment groups significantly decreased on day 1 compared to day 0 (*p* < 0.05) whereas controls did not decrease until day 2 (*p* < 0.05).

Myogenin is an MRF which is expressed following MyoD and Myf5 expression, and is necessary for the terminal differentiation of myoblasts to myotubes (Montenegro et al., 2019), so is often used as the main marker of myogenic differentiation. There was a significant day × treatment interaction (*p* < 0.001) for myogenin mRNA expression (Figure 3C). There were no significant differences in expression from day 0 to day 4, but there was an increase in expression for the 3 highest doses of 1,25(OH)_2_D_3_ on day 6 compared to day 4, although only 10^−11^ M and 10^−9^ M 1,25(OH)_2_D_3_ were significant (*p* < 0.05). Myogenin mRNA expression did not significantly increase until day 8 for controls (*p* < 0.05), at which point, expression for 10^−9^ M and 10^−7^ M 1,25(OH)_2_D_3_ was significantly lower than controls (*p* < 0.05). Hence, there was an earlier peak for myogenin expression with 1,25(OH)_2_D_3_ treatment indicating that the differentiation process had been induced sooner.

### 3.3 Vitamin D influences mRNA expression of myosin heavy chain (MHC) isoforms

**FIGURE 4:**
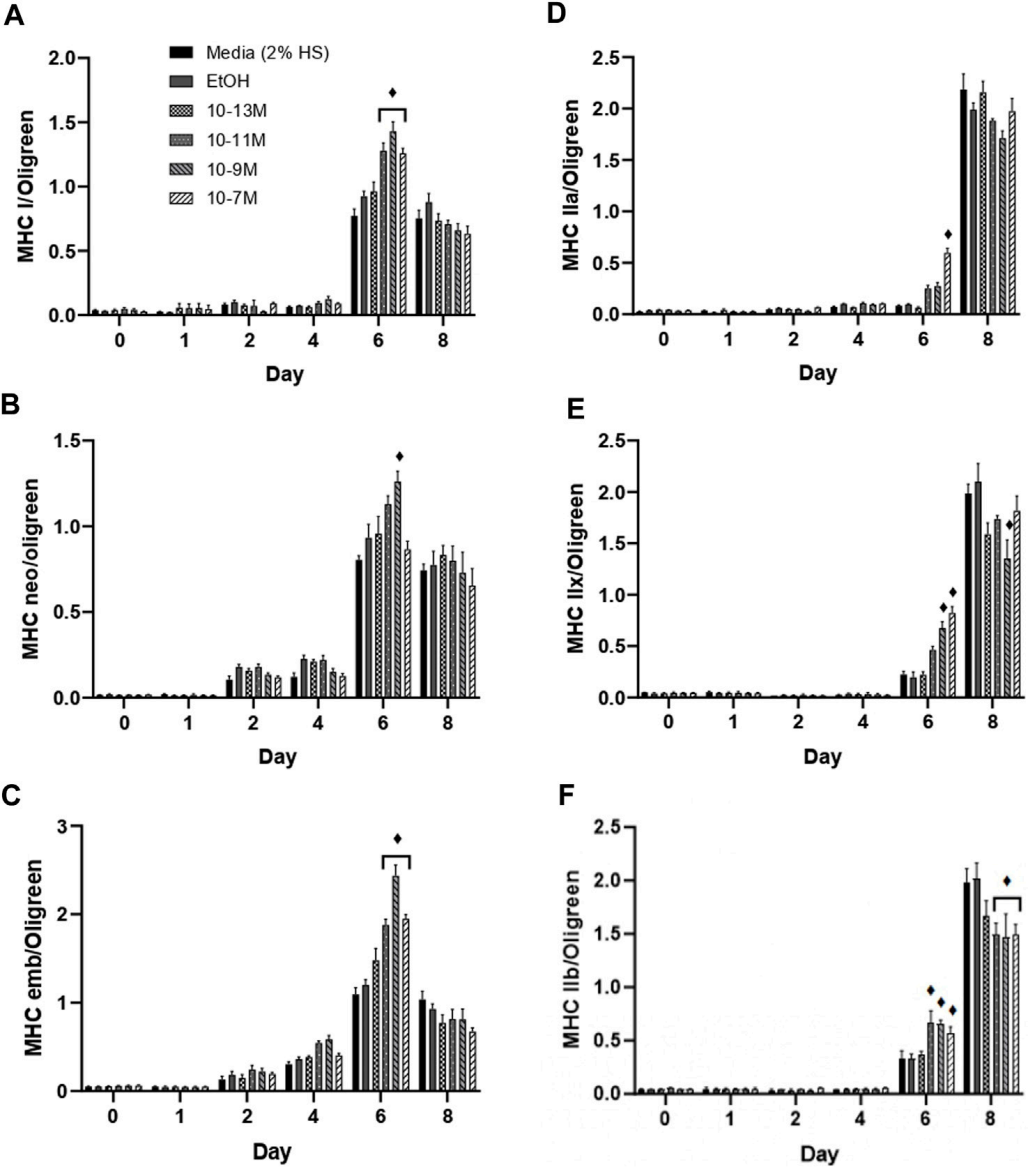
Effects of 1,25(OH)_2_D_3_ on C2C12 cells over 8 days on myosin heavy chain gene expression. **(A)** MHC I **(B)** MHC neonatal **(C)** MHC embryonic **(D)** MHC IIa **(E)** MHC IIx **(F)** MHC IIb mRNA expression normalised to Oligreen during C2C12 differentiation over an 8-day time course with varying concentrations of 1,25 (OH)_2_D_3_ dissolved in 0.1% (v/v) ethanol. ♦ symbol indicates where a bar is significantly different (*p* < 0.05) to both media and ethanol controls [media (2% HS) and EtOH] within the same time point. Error bars show standard deviation. 4 biological replicates (wells) were used throughout and all PCR experiments were carried out in triplicate. Statistical analysis was carried out via a 2-way ANOVA with a *post hoc* Bonferroni test where time*treatment interaction was significant (*p* < 0.05).

(Brown et al., 2014) showed that expression of the myosin heavy chain (MHC) isoforms can be categorised into early or late during C2C12 muscle cell differentiation. The MHC type I, neonatal and embryonic isoforms are expressed earlier during differentiation. For all 3 of these isoforms, mRNA expression remained low from day 0 to day 4, peaked on day 6, then fell on day 8 (Figures 4A–C). Importantly, on day 6, mRNA expression of both MHCI and MHC embryonic was significantly higher for the 3 highest concentrations of 1,25(OH)_2_D_3_ compared to controls (*p* < 0.05), whereas only 10^−9^ M 1,25(OH)_2_D_3_ was significantly higher than controls for MHC neonatal mRNA on day 6 (*p* < 0.05). Expression of all three early MHC isoforms was positively correlated with myogenin mRNA expression (Supplementary Data S3), with expression peaking on day 6. The strongest correlation was observed between myogenin and MHCI with an R^2^ value of 0.59 (*p* < 0.05), followed by MHC neonatal (R^2^ = 0.55, *p* < 0.05) then MHC embryonic (R^2^ = 0.49, *p* < 0.05).

The late MHC isoforms are expressed after the early isoforms in the order MHC IIa, IIx, IIb towards the latter stages of C2C12 differentiation (Brown et al., 2012), hence an increase in mRNA expression was only seen on days 6 and 8 (Figures 4D–F). MHC IIa mRNA expression was significantly increased by 10^−7^ M 1,25(OH)_2_D_3_ (*p* < 0.05), whilst MHC IIx expression was significantly increased by both 10^−9^ M and 10^−7^ M 1,25(OH)_2_D_3_ (*p* < 0.05), and MHC IIb expression was significantly increased by 10^−11^ M, 10^−9^ M and 10^−7^ M 1,25(OH)_2_D_3_ (*p* < 0.05) on day 6 compared to controls. Importantly, expression of all 3 late MHC isoforms did not change from day 0 to day 6 in the control cells, and only significantly increased on day 8 (*p* < 0.05) indicating an earlier induction of differentiation with 1,25(OH)_2_D_3_ treatment.

### 3.4 Vitamin D increases myogenin expression via a functional VDRE on the myogenin promoter

**FIGURE 5:**
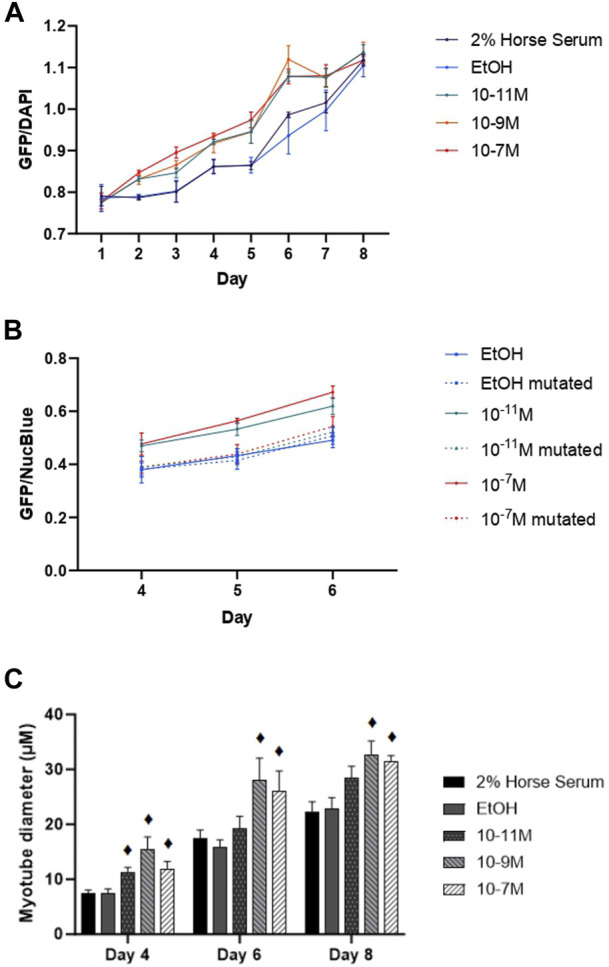
Effects of 1,25 (OH)_2_D_3_ on C2C12 cells transfected with wild-type or mutated mouse myogenin GFP vectors. **(A)** GFP fluorescence (normalised to NucBlue) using a vector containing the wild type myogenin promoter sequence 5′ to a turbo GFP sequence during C2C12 differentiation over an 8 day time course with varying concentrations of 1,25(OH)_2_D_3_ dissolved in 0.1% (v/v) ethanol. **(B)** GFP fluorescence (normalised to NucBlue) using both the wild type myogenin promoter sequence as well as the myogenin promoter sequence with a 3-base mutation in positions 2, 3, and 4 on the VDRE sequence using a 0.1% (v/v) ethanol control and two 1,25(OH)_2_D_3_ treatment concentrations (10^−7^ M and 10^−11^ M in 0.1% (v/v) ethanol) based on results from A on days 4, 5, and 6. **(C)** Myotube diameter (µM) of C2C12 cells, transfected with the wild type myogenin promoter vector 5′ to a turbo GFP sequence, grown in differentiation media with varying concentrations of 1,25(OH)_2_D_3_ dissolved in 0.1% (v/v) ethanol on days 4, 6, and 8. ♦ symbol indicates where a bar is significantly different (*p* < 0.05) to both media and ethanol controls [media (2% HS) and EtOH] within the same time point. Error bars show standard deviation and 4 biological replicates (wells) were used throughout. Statistical analysis was carried out via a 2-way ANOVA for A and C and a 3-way ANOVA for B, with a *post hoc* Bonferroni test where time*treatment interaction was significant (*p* < 0.05). There was a significant time × treatment interaction for A with GFP fluorescence for 10^−7^ M, 10^−9^ M and 10^−11^ M 1,25(OH)_2_D_3_ being 15%, 20% and 15% higher than the EtOH control on day 6 (*p* < 0.05). In B this effect of 1,25(OH)_2_D_3_ on fluorescence was lost for cells transfected with the mutated vector.

Since myogenin is a transcription factor known to induce expression of many of the genes associated with myogenic differentiation, including the MHC isoforms, we hypothesised that 1,25(OH)_2_D_3_ effects on differentiation might be via increases in mRNA myogenin expression, which subsequently increases the expression of other genes. This was backed up by the observed positive correlations between myogenin and MHC early isoform mRNA expression. We therefore investigated whether the myogenin promoter contained a putative VDRE, which was identified at −1,260 to −1,246 base pairs from the transcriptional start site, and then tested its responsiveness to 1,25(OH)_2_D_3_ by performing transfection studies using a vector containing the myogenin promoter sequence (with the putative VDRE) upstream of a GFP reporter sequence (MyoG-VDRE-GFP, Supplementary Data S2). Over the 8-day time course, there was a significant day × treatment interaction for myogenin promoter activity (*p* < 0.001, Figure 5A). On day 4, GFP fluorescence was significantly higher with 10^−7^ M and 10^−11^ M 1,25(OH)_2_D_3_ compared to controls (*p* < 0.05). By day 5, all 3 1,25(OH)_2_D_3_ treatment groups were significantly higher than controls (*p* < 0.05). The largest effects were seen on day 6, with GFP fluorescence for 10^−7^ M, 10^−9^ M, and 10^−11^ M 1,25(OH)_2_D_3_ being 15%, 20%, and 15% higher than the EtOH control (*p* < 0.05).

Transfections were then repeated using the myogenin promoter sequence with a 3-base mutation within the putative VDRE at −1,261 to −1,264 (MyoG-mutVDRE-GFP), with the MyoG-VDRE-GFP run alongside for comparison. Due to consistent results at the 3 timepoints used (days 4, 5, and 6), the day × treatment × vector interaction was not significant (*p* = 0.769, Figure 5B) but there was a significant treatment × vector interaction (*p* < 0.001). As previously observed, in cells treated with the normal VDRE construct (MyoG-VDRE-GFP), both 10^−7^ M and 10^−11^ M 1,25(OH)_2_D_3_ caused a significant increase in fluorescence compared to controls on days 4, 5, and 6 (*p* < 0.05). However, there was no effect of 1,25(OH)_2_D_3_ treatment in cells transfected with that mutated VDRE construct (MyoG-mutVDRE-GFP), indicating that responsiveness to 1,25(OH)_2_D_3_ was lost when the putative myogenin VDRE site was mutated.

Myotube diameter was measured in fluorescent myotubes and was also affected by 1,25(OH)_2_D_3_ treatment (Figure 5C). The time × treatment interaction was not significant (*p* = 0.8), but there was a significant effect of treatment (*p* < 0.001), with 10^−7^ M, 10^−9^ M and 10^−11^ M 1,25(OH)_2_D_3_ resulting in larger myotube diameters compared to controls on day 4 (*p* < 0.05), and 10^−7^ M and 10^−9^ M 1,25(OH)_2_D_3_ resulting in larger myotube diameters compared to controls on days 6 and 8 (*p* < 0.05). Cell images can be seen in Supplementary Data S4.

**FIGURE 6:**
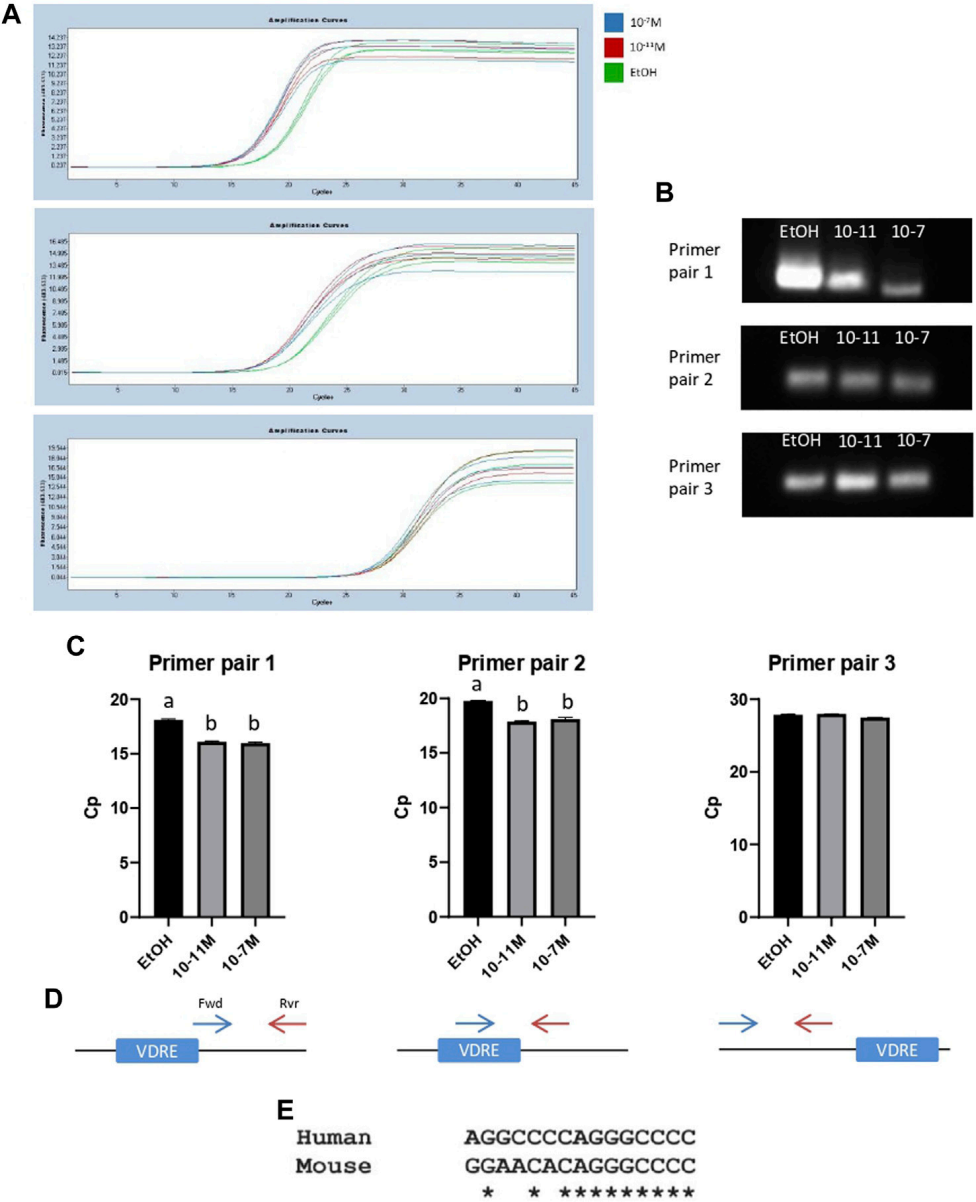
PCR products from ChIP assays for C2C12 cells treated with 1,25(OH)_2_D_3_. **(A)** PCR amplification curves for ethanol (EtOH), 10^−11^ M and 10^−7^ M 1,25(OH)_2_D_3_ generated by LightCycler 480 software following a ChIP assay using an anti-VDR antibody and primers to amplify a region of DNA around the VDRE sequence (−1,260 to −1,246). **(B)** PCR products from **(A)** run on a 2.5% (w/v) metaphor agarose gel to confirm fragment size and that a single PCR product was produced. Positive and negative controls were also run (for full gel image including ladder see Supplementary Data S5). **(C)** Average Cp values for ethanol, 10^−11^ M and 10^−7^ M 1,25(OH)_2_D_3_. Error bars show standard deviation and samples were run in triplicate. A is significantly different to b (*p* < 0.05) determined by one way ANOVA and *post hoc* Bonferroni. **(D)** Diagram to show forward (Fwd) and reverse (Rvr) primer positions in relation to the VDRE. Primer pair 1: Fwd primer starts on the last base pair of the VDRE sequence (+13 from VDRE start site), Rvr primer position is +128 from start of VDRE. Primer pair 2: Fwd primer position is +1 from start of VDRE, Rvr primer position is +41 from start of VDRE. Primer pair 3: Fwd primer position is −179 from start of VDRE, Rvr primer position is −124 from start of VDRE. **(E)** Sequence alignment using Clustal Omega between the human myogenin promoter sequence (Accession AF050501.1) and the newly identified mouse myogenin VDRE sequence.

To further confirm that the identified VDRE sequence was functional, and that the VDR binds to this region, a ChIP assay was performed. Following the pull-down of genomic DNA fragments using a VDR-specific antibody, 3 different primer pairs were used for PCR to amplify slightly different regions of DNA around the VDRE. Amplicon lengths for primer pairs 1, 2, and 3 were 138 bp, 62 bp, and 82 bp respectively (Supplementary Data S1). Amplification curves are shown in Figures 6A a single PCR amplicon was produced for all 3 primer pairs and melt curve analysis also showed a single peak, again indicating a single product. The PCR products were run on a 2.5% metaphor agarose gel to confirm that they were the correct length (Figure 6B; Supplementary Data S5). For primer pairs 1 and 2 (which both spanned the VDRE), the Cp values for 1,25(OH)_2_D_3_ treated cells were significantly lower than the control (*p* < 0.05, Figure 6C) indicating an increase in binding of the VDR to the VDRE with 1,25(OH)_2_D_3_ treatment. However, Cp values for primer pair 3 were not significantly different (Figure 6C), likely because they bind ∼97 bp upstream of the VDRE (Figure 6D) and therefore some DNA fragments containing the VDRE were not detected by this primer pair.

When the newly identified mouse myogenin VDRE sequence was aligned to the human myogenin genomic sequence (Accession AF050501.1) using Clustal Omega, it revealed an 11/15 (73%) base pair match (Figure 6E). This could indicate that this VDRE sequence on the myogenin promoter is conserved across species. However, alignment with cow, pig and sheep myogenin sequences was attempted but not possible due to the available sequences not containing enough of the 3’ promoter.

## 4 Discussion

For the first time, this detailed C2C12 differentiation time course study has shown a clear stimulatory effect of active VD [1,25(OH)_2_D_3_] on myogenic differentiation which appears to be via a functional VDRE on the myogenin gene promoter.

Firstly, we showed that 1,25(OH)_2_D_3_ inhibits cell proliferation, inducing a switch towards a differentiative state. At 48 h, DNA content for the highest two 1,25(OH)_2_D_3_ concentrations were similar to day 0 indicating that the reduced level of DNA compared to controls was due to an inhibition of proliferation rather than an increase in apoptosis. This agrees with previous work in C2C12 cells showing a 75% decrease in proliferating cell nuclear antigen (PCNA), a marker of proliferation, after 7 days in 1,25(OH)_2_D_3_ treated cells (Garcia et al., 2011), and also agrees with work in chick myoblasts where proliferation was significantly decreased by 1,25(OH)_2_D_3_ on days 4 and 6 compared to controls (Capiati et al., 1999). It has been reported that myoblasts treated with active VD have a higher percentage of cells in the G_0_/G_1_ (quiescent) phase and a lower percentage of cells in the S/M (active) phase compared to controls (Girgis et al., 2014) suggesting that this anti-proliferative effect of VD is associated with cell cycle arrest. Our results show that creatine kinase (CK) activity, which is expressed after myoblasts exit from the cell cycle and begin to differentiate (Tai et al., 2011), is significantly higher for 1,25(OH)_2_D_3_ treated cells than controls. Again, this agrees with (Capiati et al., 1999) who showed that CK activity was increased in 1,25(OH)_2_D_3_ treated chick myoblasts on days 2, 4 and, 6 compared to controls, which suggests that active VD inhibits proliferation and stimulates cells to differentiate.

The MRFs are basic helix-loop-helix transcription factors that recognise and bind to E box sequences (CANNTG) on target genes (Adhikari et al., 2021). They possess potent signaling effects, such that inducing expression of any of the MRFs on a variety of tissue culture cells results in conversion to muscle-type cells (Hasty et al., 1993). Myf5 is the first MRF to be expressed, followed closely by MyoD and then myogenin (Zammit, 2017). The myogenin promoter contains E box sequences both within the proximal promoter sequence (−130 to +18 bp) and in enhancer regions −4.5 kb and −6.5 kb upstream of the coding sequence, which become bound by MyoD/E-protein heterodimers to activate transcription during differentiation (Faralli and Dilworth, 2012). Our results show that Myf5 mRNA expression is significantly lower in 1,25(OH)_2_D_3_ treated cells than controls on day 1, possibly indicating a faster down regulation of early differentiation markers, which may suggest that cells are differentiating more quickly. These results are in agreement with previous work which found a decrease in Myf5 gene expression just 6 h post 1,25(OH)_2_D_3_ injection in chick embryos (Chen et al., 2021). In addition, work in C2C12 cells showed that both inactive [25(OH)D_3_] and active VD treatment reduced Myf5 mRNA expression (Girgis et al., 2014). MyoD mRNA expression was not found to be affected by 1,25(OH)_2_D_3_ in this study. The current literature reports inconsistent effects of active VD on MyoD mRNA expression *in vitro* with some finding increased expression (Garcia et al., 2011; Saito et al., 2017), one study finding decreased expression (Olsson et al., 2016) and another finding no significant effect (van der Meijden et al., 2016). It is worth noting that there is large variability between these studies on the day that MyoD expression was measured, often at only a single time point, which ranged from day 1 to day 7. Despite this study finding no change in MyoD mRNA expression, it is worth noting that MyoD increases transcription of p21 which inhibits various cyclin dependent kinases (CDKs), thus leading to cell cycle arrest, allowing myoblasts to enter a differentiative state (Christ and Brand-Saberi, 2002). Previous studies have shown an increase in p21 mRNA expression in response to vitamin D (Okuno et al., 2012; Olsson et al., 2016), therefore it cannot be ruled out that vitamin D could also be acting via cell cycle regulatory factors to stimulate cells to enter the differentiation phase more quickly. As far as we are aware, this study has used the most detailed time course to measure the effects of 1,25(OH)_2_D_3_ on MRF expression, measuring at 6 different time points across differentiation. Our study shows that 1,25(OH)_2_D_3_ causes an earlier increase in myogenin mRNA expression compared to controls. There are also inconsistencies within the literature on the effect of active VD on myogenin expression *in vitro* with some studies finding a decrease in mRNA expression (Okuno et al., 2012; Ryan et al., 2013; Girgis et al., 2014; Olsson et al., 2016; van der Meijden et al., 2016) and others finding an increase in mRNA expression (Garcia et al., 2011; Gili et al., 2016; Braga et al., 2017; Montenegro et al., 2019), and protein expression (2, 36). Again, most of these studies measure expression at one or two time points, rather than using time courses, with variation on day of measurement. *In vivo*, dietary VD [25(OH)D_3_] has been shown to increase myogenin expression in piglets from mothers fed a high VD diet, with higher levels of myogenin mRNA observed in the longissimus dorsi and psoas major muscles compared to controls (Zhou et al., 2016).

Myogenin is an essential component of the regulatory pathway that leads to skeletal muscle formation during embryogenesis. In myogenin knockout mice, pups survive fetal development but are born immobile and die shortly after birth with a lack of differentiated skeletal muscle, despite normal levels of MyoD (Hasty et al., 1993). In contrast, knockout of either MyoD or Myf5 result in normal muscle formation (Asfour et al., 2018), but knockout of both Myf5 and MyoD results in complete lack of skeletal muscle (Rudnicki et al., 1993), with mice being born alive but immobile, and dying soon after birth. A previous study has shown that myogenin mRNA expression is completely blocked when the VDR is knocked down *in vitro* (Gili et al., 2016) suggesting that VD is essential for myogenin expression. The VDRE sequence that we have found is, to our knowledge, the first time that a functional VDRE has been identified on the myogenin promoter. Our results show that when this VDRE sequence is mutated, the increase in myogenin mRNA expression previously seen in 1,25(OH)_2_D_3_ treated cells becomes abolished, suggesting that this sequence is essential for the upregulation of myogenin gene transcription by 1,25(OH)_2_D_3_. In addition to this, ChIP analysis showed that the VDR does in fact bind to the sequence identified suggesting that the effects of 1,25(OH)_2_D_3_ on myogenin mRNA expression are direct and VDR mediated.

A recent study has shown that depleting myogenin levels via shRNA in C2C12 cells resulted in a significant reduction in myosin heavy chain protein levels (Adhikari et al., 2021). In addition, over expression of rat myogenin during mouse fetal development results in increased levels of oxidative enzymes within skeletal muscle (Hughes et al., 1999). Both of these suggest that myogenin plays a role in regulating expression of the slow twitch, or early, MHC isoforms which are oxidative in nature. In agreement with this, our results show a positive correlation between myogenin and early MHC isoform mRNA expression. Therefore, the increase in mRNA expression of the early MHC isoforms observed in response to 1,25(OH)_2_D_3_ is likely to be due, at least in part, to the increase in myogenin expression.

Our previous work has found that during C2C12 differentiation the MHC fast isoforms are expressed in the order IIa, IIx, and IIb (Brown et al., 2012). Our results show an earlier increase in expression of all three of the fast isoforms with 1,25(OH)_2_D_3_ compared to controls, suggesting that treatment causes cells to move through differentiation more quickly. In agreement, other work has found that eldecalcitol (a VD analogue) increased mRNA expression of all three fast isoforms in C2C12 cells (Saito et al., 2017). In addition to this, one study found that 1,25(OH)_2_D_3_ caused an increase both MHC IIa mRNA and protein expression in already differentiated C2C12 cells (Okuno et al., 2012) suggesting that the effects of VD on MHC expression may not be isolated to embryogenesis, but may also have effects in differentiated muscle.

Finally, we show that 1,25(OH)_2_D_3_ causes a significant increase in myotube diameter. In our recent systematic review (Alliband et al., 2021) there was strong agreement across the literature with all 5 *in vitro* studies that measured myotube size finding a significant increase with 1,25(OH)_2_D_3_. This is reflected *in vivo* since rat pups from VD deficient mothers had smaller muscle fibers and larger interfibrillar spaces within the muscle (Max et al., 2014). Cluster gene analysis showed that VD sufficient pups showed an upregulation of genes involved in cell differentiation and a down regulation of genes involved in protein catabolism compared to VD deficient pups (Max et al., 2014). In addition, piglets born to mothers fed a high VD diet show a larger muscle fiber cross sectional area in the psoas major and longissimus dorsi muscles (Zhou et al., 2016). Hence, there appear to be a clear effects of VD on muscle fiber size both *in vitro* and *in vivo*.

Overall, the results presented show that active VD directly increases myogenin transcription via a functional VDRE on the myogenin promoter, resulting in increased myogenic differentiation, increased mRNA expression of both the early and late MHC isoforms, as well as increased myotube size. However, we cannot rule out that 1,25(OH)_2_D_3_ may also be exerting direct effects on expression of the MHC isoforms. Future work could investigate the effects of VD at the protein level to check that the effects on gene expression found in this study correlate with effects at the protein level. Future work also should investigate the impact of VD deficiency during embryogenesis *in vivo,* in relation to muscle fibre development, MRF and MHC expression in the muscle of the offspring, as well as the long term impact on postnatal muscle growth and body composition. Finally, an 11/15 base pair match was found between the mouse myogenin VDRE sequence and a potential VDRE sequence on the human myogenin genomic sequence. Therefore, further work to investigate the impact of 1,25(OH)_2_D_3_ on human myogenesis is needed. Currently, it is estimated that between 20%-40% of pregnant women worldwide are vitamin D insufficient or deficient (Urrutia-Pereira and Solé, 2015), therefore it is crucial that a clearer understanding of the impact of maternal VD deficiency on offspring development and health is gained.

## Data Availability

The original contributions presented in the study are included in the article/Supplementary Material, further inquiries can be directed to the corresponding author.

## Abbreviations

VD, vitamin D; VDR, vitamin D receptor; RAR, retinoid acid receptor; RXR, retinoid X receptor; VDRE, vitamin D response element; MRF, myogenic regulatory factor; MHC, myosin heavy chain; CK, creatine kinase; GFP, green fluorescent protein.

## References

[B1] Adhikari A. Kim W. Davie J. (2021). Myogenin is required for assembly of the transcription machinery on muscle genes during skeletal muscle differentiation. PLoS One 16, e0245618. 10.1371/journal.pone.0245618 33465133 PMC7815108

[B2] Alliband K. H. Kozhevnikova S. V. Parr T. Jethwa P. H. Brameld J. M. (2021). *In vitro* effects of biologically active vitamin D on myogenesis: a systematic review. Front. Physiol. 12, 736708. 10.3389/fphys.2021.736708 34566700 PMC8458760

[B3] Asfour H. A. Allouh M. Z. Said R. S. (2018). Myogenic regulatory factors: the orchestrators of myogenesis after 30 years of discovery. Exp. Biol. Med. (Maywood) 243, 118–128. 10.1177/1535370217749494 29307280 PMC5788151

[B4] Braga M. Simmons Z. Norris K. C. Ferrini M. G. Artaza J. N. (2017). Vitamin D induces myogenic differentiation in skeletal muscle derived stem cells. Endocr. Connect. 6, 139–150. 10.1530/EC-17-0008 28174253 PMC5424772

[B5] Brearley M. C. Loczenski-Brown D. M. Loughna P. T. Parr T. Brameld J. M. (2021). Response of the porcine *MYH4*-promoter and *MYH4*-expressing myotubes to known anabolic and catabolic agents *in vitro* . Biochem. Biophys. Rep. 25, 100924. 10.1016/j.bbrep.2021.100924 33614996 PMC7880916

[B6] Brown D. M. Brameld J. M. Parr T. (2014). Expression of the myosin heavy chain IIB gene in porcine skeletal muscle: the role of the CArG-Box promoter response element. PLoS One 9, e114365. 10.1371/journal.pone.0114365 25469802 PMC4255089

[B7] Brown D. M. Jones S. Daniel Z. C. T. R. Brearley M. C. Lewis J. E. Ebling F. J. P. (2018). Effect of sodium 4-phenylbutyrate on Clenbuterol-mediated muscle growth. PLoS One 13, e0201481. 10.1371/journal.pone.0201481 30052661 PMC6063449

[B8] Brown D. M. Parr T. Brameld J. M. (2012). Myosin heavy chain mRNA isoforms are expressed in two distinct cohorts during C2C12 myogenesis. J. Muscle Res. Cell Motil. 32, 383–390. 10.1007/s10974-011-9267-4 22012579

[B9] Capiati D. A. Téllez-Iñón M. T. Boland R. L. (1999). Participation of protein kinase C alpha in 1,25-dihydroxy-vitamin D3 regulation of chick myoblast proliferation and differentiation. Mol. Cell Endocrinol. 153, 39–45. 10.1016/s0303-7207(99)00093-3 10459852

[B10] Carlberg C. (2019). Nutrigenomics of vitamin D. Nutrients 11, 676. 10.3390/nu11030676 30901909 PMC6470874

[B11] Carlberg C. Campbell M. J. (2013). Vitamin D receptor signaling mechanisms: integrated actions of a well-defined transcription factor. Steroids 78, 127–136. 10.1016/j.steroids.2012.10.019 23178257 PMC4668715

[B12] Carlberg C. Molnár F. (2015). Vitamin D receptor signaling and its therapeutic implications: genome-wide and structural view. Can. J. Physiol. Pharmacol. 93, 311–318. 10.1139/cjpp-2014-0383 25741777

[B13] Chen C. White D. L. Marshall B. Kim W. K. (2021). Role of 25-hydroxyvitamin D_3_ and 1,25-dihydroxyvitamin D_3_ in chicken embryo osteogenesis, adipogenesis, myogenesis, and vitamin D_3_ metabolism. Front. Physiol. 12, 637629. 10.3389/fphys.2021.637629 33597896 PMC7882680

[B14] Christakos S. Dhawan P. Verstuyf A. Verlinden L. Carmeliet G. (2016). Vitamin D: metabolism, molecular mechanism of action, and pleiotropic effects. Physiol. Rev. 96, 365–408. 10.1152/physrev.00014.2015 26681795 PMC4839493

[B15] Christ B. Brand-Saberi B. (2002). Limb muscle development. Int. J. Dev. Biol. 46, 905–914. 10.1387/ijdb.12455629 12455628

[B16] Endo I. Inoue D. Mitsui T. Umaki Y. Akaike M. Yoshizawa T. (2003). Deletion of vitamin D receptor gene in mice results in abnormal skeletal muscle development with deregulated expression of myoregulatory transcription factors. Endocrinology 144, 5138–5144. 10.1210/en.2003-0502 12959989

[B17] Faralli H. Dilworth F. J. (2012). Turning on myogenin in muscle: a paradigm for understanding mechanisms of tissue-specific gene expression. Comp. Funct. Genomics 2012, 836374. 10.1155/2012/836374 22811619 PMC3395204

[B18] Garcia L. A. King K. K. Ferrini M. G. Norris K. C. Artaza J. N. (2011). 1,25(OH)2vitamin D3 stimulates myogenic differentiation by inhibiting cell proliferation and modulating the expression of promyogenic growth factors and myostatin in C2C12 skeletal muscle cells. Endocrinology 152, 2976–2986. 10.1210/en.2011-0159 21673099 PMC3138228

[B19] Gili V. Pardo V. G. Ronda A. C. De Genaro P. Bachmann H. Boland R. (2016). *In vitro* effects of 1α,25(OH)₂D₃-glycosides from Solbone A (Solanum glaucophyllum leaves extract; Herbonis AG) compared to synthetic 1α,25(OH)₂D₃ on myogenesis. Steroids 109, 7–15. 10.1016/j.steroids.2016.03.002 26968127

[B20] Girgis C. M. Clifton-Bligh R. J. Mokbel N. Cheng K. Gunton J. E. (2014). Vitamin D signaling regulates proliferation, differentiation, and myotube size in C2C12 skeletal muscle cells. Endocrinology 155, 347–357. 10.1210/en.2013-1205 24280059

[B21] Griffin G. Hewison M. Hopkin J. Kenny R. A. Quinton R. Rhodes J. (2021). Preventing vitamin D deficiency during the COVID-19 pandemic: UK definitions of vitamin D sufficiency and recommended supplement dose are set too low. Clin. Med. (Lond) 21, e48–e51. 10.7861/clinmed.2020-0858 33158957 PMC7850219

[B22] Hasty P. Bradley A. Morris J. H. Edmondson D. G. Venuti J. M. Olson E. N. (1993). Muscle deficiency and neonatal death in mice with a targeted mutation in the myogenin gene. Nature 364, 501–506. 10.1038/364501a0 8393145

[B23] Hughes S. M. Chi M. M. Lowry O. H. Gundersen K. (1999). Myogenin induces a shift of enzyme activity from glycolytic to oxidative metabolism in muscles of transgenic mice. J. Cell Biol. 145, 633–642. 10.1083/jcb.145.3.633 10225962 PMC2185087

[B24] Hurley M. S. Flux C. Salter A. M. Brameld J. M. (2006). Effects of fatty acids on skeletal muscle cell differentiation *in vitro* . Br. J. Nutr. 95, 623–630. 10.1079/bjn20051711 16512949

[B25] Kitzmann M. Fernandez A. (2001). Crosstalk between cell cycle regulators and the myogenic factor MyoD in skeletal myoblasts. Cell. Mol. Life Sci. 58, 571–579. 10.1007/PL00000882 11361092 PMC11146557

[B26] Max D. Brandsch C. Schumann S. Kühne H. Frommhagen M. Schutkowski A. (2014). Maternal vitamin D deficiency causes smaller muscle fibers and altered transcript levels of genes involved in protein degradation, myogenesis, and cytoskeleton organization in the newborn rat. Mol. Nutr. Food Res. 58, 343–352. 10.1002/mnfr.201300360 23963738

[B27] Molnár F. (2014). Structural considerations of vitamin D signaling. Front. Physiol. 5, 191. 10.3389/fphys.2014.00191 24936188 PMC4048014

[B28] Montenegro K. R. Cruzat V. Carlessi R. Newsholme P. (2019). Mechanisms of vitamin D action in skeletal muscle. Nutr. Res. Rev. 32, 192–204. 10.1017/S0954422419000064 31203824

[B29] Okuno H. Kishimoto K. N. Hatori M. Itoi E. (2012). 1α,25-dihydroxyvitamin D₃ enhances fast-myosin heavy chain expression in differentiated C2C12 myoblasts. Cell Biol. Int. 36, 441–447. 10.1042/CBI20100782 22276695

[B30] Olsson K. Saini A. Strömberg A. Alam S. Lilja M. Rullman E. (2016). Evidence for vitamin D receptor expression and direct effects of 1α,25(OH)2D3 in human skeletal muscle precursor cells. Endocrinology 157, 98–111. 10.1210/en.2015-1685 26469137

[B31] Pagana K. D. Pagana T. J. Pagana T. N. (2019). Mosby’s diagnostic & laboratory test reference. St Louis: Elsevier Mosby.

[B32] Rago R. Mitchen J. Wilding G. (1990). DNA fluorometric assay in 96-well tissue culture plates using Hoechst 33258 after cell lysis by freezing in distilled water. Anal. Biochem. 191, 31–34. 10.1016/0003-2697(90)90382-j 1706565

[B33] Rudnicki M. A. Schnegelsberg P. N. Stead R. H. Braun T. Arnold H. H. Jaenisch R. (1993). MyoD or Myf-5 is required for the formation of skeletal muscle. Cell 75, 1351–1359. 10.1016/0092-8674(93)90621-v 8269513

[B34] Ryan K. J. Daniel Z. C. Craggs L. J. Parr T. Brameld J. M. (2013). Dose-dependent effects of vitamin D on transdifferentiation of skeletal muscle cells to adipose cells. J. Endocrinol. 217, 45–58. 10.1530/JOE-12-0234 23328072 PMC3600570

[B35] Saito H. Kishimoto K. N. Mori Y. Okuno H. Tanaka M. Itoi E. (2017). A vitamin D analogue, eldecalcitol, enhances expression of fast myosin heavy chain subtypes in differentiated C2C12 myoblasts. J. Orthop. Sci. 22, 345–350. 10.1016/j.jos.2016.12.005 28017710

[B36] Tai P. W. Fisher-Aylor K. I. Himeda C. L. Smith C. L. Mackenzie A. P. Helterline D. L. (2011). Differentiation and fiber type-specific activity of a muscle creatine kinase intronic enhancer. Skelet. Muscle 1, 25. 10.1186/2044-5040-1-25 21797989 PMC3157005

[B37] Thompson P. D. Jurutka P. W. Haussler C. A. Whitfield G. K. Haussler M. R. (1998). Heterodimeric DNA binding by the vitamin D receptor and retinoid X receptors is enhanced by 1,25-dihydroxyvitamin D3 and inhibited by 9-cis-retinoic acid. Evidence for allosteric receptor interactions. J. Biol. Chem. 273, 8483–8491. 10.1074/jbc.273.14.8483 9525962

[B38] Tsoumpra M. K. Sawatsubashi S. Imamura M. Fukumoto S. Takeda S. Matsumoto T. (2020). Dystrobrevin alpha gene is a direct target of the vitamin D receptor in muscle. J. Mol. Endocrinol. 64, 195–208. 10.1530/JME-19-0229 31940280

[B39] Urrutia-Pereira M. Solé D. (2015). Vitamin D deficiency in pregnancy and its impact on the fetus, the newborn and in childhood. Rev. Paul. Pediatr. 33, 104–113. 10.1016/j.rpped.2014.05.004 25662013 PMC4436962

[B40] Van Der Meijden K. Bravenboer N. Dirks N. F. Heijboer A. C. Den Heijer M. De Wit G. M. (2016). Effects of 1,25(OH)2 D3 and 25(OH)D3 on C2C12 myoblast proliferation, differentiation, and myotube hypertrophy. J. Cell Physiol. 231, 2517–2528. 10.1002/jcp.25388 27018098 PMC5111790

[B41] Wang T. Tavera-Mendoza L. Laperriere D. Libby E. Macleod N. Nagai Y. (2005). Large-scale *in silico* and microarray-based identification of direct 1,25-dihydroxyvitamin D3 target genes. Mol. Endocrinol. 19, 2685–2695. 10.1210/me.2005-0106 16002434

[B42] Zammit P. S. (2017). Function of the myogenic regulatory factors Myf5, MyoD, Myogenin and MRF4 in skeletal muscle, satellite cells and regenerative myogenesis. Semin. Cell Dev. Biol. 72, 19–32. 10.1016/j.semcdb.2017.11.011 29127046

[B43] Zhou H. Chen Y. Lv G. Zhuo Y. Lin Y. Feng B. (2016). Improving maternal vitamin D status promotes prenatal and postnatal skeletal muscle development of pig offspring. Nutrition 32, 1144–1152. 10.1016/j.nut.2016.03.004 27209214

