# Supplementary material for: Active vitamin D increases myogenic differentiation in C2C12 cells via a vitamin D response element on the myogenin promoter
Source: Front Physiol. 2024 Jan 8;14:1322677. doi: 10.3389/fphys.2023.1322677 (PMC10804454; doi:10.3389/fphys.2023.1322677)

Supplementary Material

Active vitamin D increases myogenic differentiation in C2C12 cells via a vitamin D response element on the myogenin promoter

Kathryn H. Alliband, Tim Parr, Preeti H. Jethwa, John M. Brameld*

Division of Food, Nutrition and Dietetics, School of Biosciences, University of Nottingham, Sutton Bonington Campus, Loughborough, LE12 5RD, United Kingdom.

*** Correspondence:**
Corresponding Author: John Brameld
john.brameld@nottingham.ac.uk

Supplementary data 1 – List of primer sequences

Supplementary data 1 shows a table with a list of forward and reverse primer sequences, and amplicon length, for all PCR primers to measure gene expression and to amplify DNA fragments from ChIP.

Target Transcript	*Forward primer sequence*	*Reverse primer sequence*	*Length of amplicon*
MyoD	5′ CGTGGCAGCGAGCACTAC 3′	5′ TGTAATCCATCATGCCATCAGA 3′	79bp
Myf5	5′ CAGCCCCACCTCCAACTG 3′	5′ GCAGCACATGCATTTGATACATC 3′	117bp
Myogenin	5′ CCCATGGTGCCCAGTGAA 3′	5′ GCAGATTGTGGGCGTCTGTA 3′	130bp
Myosin Heavy Chain I (MHCI)	5′ CTCAAGCTGCTCAGCAATCTATTT 3′	5′ GGAGCGCAAGTTTGTCATAAGT 3′	152bp
Myosin Heavy Chain neonatal (MHC neo)	5′ CAGGAGCAGGAATGATGCTCTGAG 3′	5′ AGTTCCTCAAACTTTCAGCAGC CAA 3′	113bp
Myosin Heavy Chain embryonic (MHC emb)	5′ TCCGACAACGCCTACCAGTT 3′	5′ CCCGGATTCTCCGGTGAT 3′	72bp
Myosin Heavy Chain IIa	5′ AGGCGGCTGAGGAGCACGTA 3′	5′ GCGGCACAAGCAGCGTTGG 3′	130bp
Myosin Heavy Chain IIx	5′ GAGGGACAGTTCATCGATAGCAA 3′	5′ GGGCCAACTTGTCATCTCTCAT 3′	154bp
Myosin Heavy Chain IIb	5′ CAATCAGGAACCTTCGGAACAC 3′	5′ GTCCTGGCCTCTGAGAGCAT 3′	80bp
VDRE ChIP primer 1	5′ CCCAATGGAGGAGCTAGAGAAA 3′	5′ CGACTAGGCCATCTTCTGCTACA 3′	138bp
VDRE ChIP primer 2	5′ GAACACAGGGCCCCCAAT 3′	5′ TGCAGACCCCTTCAGTTCCTT 3′	62bp
VDRE ChIP primer 3	5′ CACAGAAGAGATGATTAAGAGC ATCAG 3′	5′ GCTGGCATATGTAATAGTGTCT GTGTT 3′	82bp

Supplementary data 2 – vector sequence and components

Supplementary data 2 shows a breakdown of components and the full genetic sequence for both vectors used in the transfection experiments (MyoG-VDRE-GFP and MyoG-mutVDRE-GFP). The VDRE sequence is highlighted in yellow and the 3 base mutation is shown in red. The rest of the sequence is colour coded (underlined) according to the corresponding vector table.

Vector name – **MyoG-VDRE-GFP**

Name	Position	Size (bp)	Type	Description	Application notes
Myogenin promoter including normal VDRE	1-1577	1577	Promoter	Promoter sequence for mouse gene Myogenin	Region of DNA upstream from the Myogenin coding sequence where relevant proteins bind to initiate or repress transcription of the gene.
Kozak	1608-1613	6	Miscellaneous	Kozak translation initiation sequence	Facilitates translation initiation of ATG start codon downstream of the Kozak sequence.
TurboGFP	1614-2297	684	ORF	Also known as maxGFP; green fluorescent protein from maxillopoda	Fast maturation; ranked high in brightness, photostability and pH stability among all fluorescent proteins.
SV40 late pA	2398-2619	222	PolyA_signal	Simian virus 40 late polyadenylation signal	Allows transcription termination and polyadenylation of mRNA transcribed by Pol II RNA polymerase.
pUC ori	complement (2815-3403)	589	Rep_origin	pUC origin of replication	Facilitates plasmid replication in E. coli; regulates high-copy plasmid number (500-700).
Ampicillin	complement (3574-4434)	861	ORF	Ampicillin resistance gene	Allows E. coli to be resistant to ampicillin.

Sequence:

1 TCTAGAGTTG TATGACGCAG GCAAAGGTGA TGACTCAGGC AGGAAGGAAT AGAAGAGGCC AGCCTGGTGG CCCAGGACAG
81 ACAAATGATG CAAAGGACTC TTTCTCCTTA TCGACCCTTC TACAGAAAGG AAAGAGTCAA AACGGTTCTA GTGCCAGAAG
161 GCATTATTGA GGGGAAAGCA CAGAAGAGAT GATTAAGAGC ATCAGACAGG GTCCATCCCA TAATCAGCCA CCAAACACAG
241 ACACTATTAC ATATGCCAGC AAGATTTTGC TGAAAGAACC CTGATATAGC TGCCTCTTGT GAGGCTATGC AGTGCCTGGC
321 AAATACAGAA GTGGATGCTC ACAGTCAGCT ATTGGATGGA ACACAGGGCC CCCAATGGAG GAGCTAGAGA AAGTACCCAA

401 GGAACTGAAG GGGTCTGCAA CCCTATAGGT GGAACAACAA TATGAACTAA CCAGTACCCC CAGAGCTCAT GTCTCTAGCT

481 GCATATGTAG CAGAAGATGG CCTAGTCGGC CATCATTGGG AAGAGAGGCC CCTTGGTATT GCAAACTATA TGCCCCAGTA

561 CAGGGGAACG CCAGGGCCAA GAAGTGGGAA TGAGTGGGTA GGGGAGCAGG GCGGGGGGAG GGGGGGGTAT AGGGAACTTT

641 TGGGATAGCA TTTGAAATGT AAATGAAGAA AATATCTAAT AAAAAATAAT TTAAAAAAGA GCGTCAGACA GGGGACTGAA

721 CAGCTCTTGC ACTAGGGGAG AAGAAGGCAA TGTAGAGTAG TCTGTGAGTT CTAATCCTTG CTAAACACTG ACTTCACCTG

801 ACCCCTACTA CTTAAGGCCC CCCCCCCTTA CTTAAGAAGT CCCTGTGTTC TCTTACTTCA ATCTACCCCC AACATCATGA

881 GACCTGGTCA AAGAAGCTGT AGAAACCCAA AAGTTGAATC CATTTGCCCT TCTGGGTTTC TGTCTTTGCC TCCATGGACG

961 ATAGGACACA CACACACACA CACACACACA CACACACACA CACACACACA CGCCCCAAAT CTGGAGTGGT CCTGATGTGG

1041 TAGTGGTAGG TCTTTAGGGG TCTCATGGGA CTGACATAGT ACGGTTTAAG GTGCTGCTGC TGAGCAGGAA AGAGAAGGCT

1121 AAGTGGATTT TCAAGACCCC TTCCCGTCCG TCCAAGACAA CCCCTTTCTT GTTCCCTTCC TGCCCTGTCC ACCAGCTGCC

1201 TTGGACCATG GAGGAGAGAG TAGGCAGGAG GCCCGGGTAG GAGTAATTGA AAGGAGCAGA TGAGACGGGG GAATGCACCC

1281 ACCCCCACCT TCCCTGCCCC ACAGGCTGTG GAGAAATGAA AACTAATCAA ATTACAGCCG ACGGCCTCCC GACCCGTGCA

1361 CAGGAGCCGC CTGGGCCAGG GGCAGGCCTG CAGGGTGGGG TGGGGGCAAA AGGAGAGGGA AGGGGAATCA CATGTAATCC

1441 ACTGGAAACG TCTTGATGTG CAGCAACAGC TTAGAGGGGG GCTCAGGTTT CTGTGGCGTT GGCTATATTT ATCTCTGGGT

1521 TCATGCCAGC AGGGAGGGTT TAAATGGCAC CCAGCAGTTG GCGTGAGGGG CTGCGGGTCT AGACAAGTTT GTACAAAAAA

1601 GCAGGCTGCC ACCATGCCCG CCATGAAGAT CGAGTGCCGC ATCACCGGCA CCCTGAACGG CGTGGAGTTC GAGCTGGTGG

1681 GCGGCGGAGA GGGCACCCCC GAGCAGGGCC GCATGACCAA CAAGATGAAG AGCACCAAAG GCGCCCTGAC CTTCAGCCCC

1761 TACCTGCTGA GCCACGTGAT GGGCTACGGC TTCTACCACT TCGGCACCTA CCCCAGCGGC TACGAGAACC CCTTCCTGCA

1841 CGCCATCAAC AACGGCGGCT ACACCAACAC CCGCATCGAG AAGTACGAGG ACGGCGGCGT GCTGCACGTG AGCTTCAGCT

1921 ACCGCTACGA GGCCGGCCGC GTGATCGGCG ACTTCAAGGT GGTGGGCACC GGCTTCCCCG AGGACAGCGT GATCTTCACC

2001 GACAAGATCA TCCGCAGCAA CGCCACCGTG GAGCACCTGC ACCCCATGGG CGATAACGTG CTGGTGGGCA GCTTCGCCCG

2081 CACCTTCAGC CTGCGCGACG GCGGCTACTA CAGCTTCGTG GTGGACAGCC ACATGCACTT CAAGAGCGCC ATCCACCCCA

2161 GCATCCTGCA GAACGGGGGC CCCATGTTCG CCTTCCGCCG CGTGGAGGAG CTGCACAGCA ACACCGAGCT GGGCATCGTG

2241 GAGTACCAGC ACGCCTTCAA GACCCCCATC GCCTTCGCCA GATCTCGAGC TCGATGACGC ACCAAGGAAG CCCTCGAGGA

2321 CGCGTAAAGG TACCAAAGGA TCCCGACCTA CCGACCCAGC TTTCTTGTAC AAAGTGGTGA TGGCCGGCCG CTTCGAGCAG

2401 ACATGATAAG ATACATTGAT GAGTTTGGAC AAACCACAAC TAGAATGCAG TGAAAAAAAT GCTTTATTTG TGAAATTTGT

2481 GATGCTATTG CTTTATTTGT AACCATTATA AGCTGCAATA AACAAGTTAA CAACAACAAT TGCATTCATT TTATGTTTCA

2561 GGTTCAGGGG GAGGTGTGGG AGGTTTTTTA AAGCAAGTAA AACCTCTACA AATGTGGTAG CGGCCGCGGC GCTCTTCCGC

2641 TTCCTCGCTC ACTGACTCGC TGCGCTCGGT CGTTCGGCTG CGGCGAGCGG TATCAGCTCA CTCAAAGGCG GTAATACGGT

2721 TATCCACAGA ATCAGGGGAT AACGCAGGAA AGAACATGTG AGCAAAAGGC CAGCAAAAGG CCAGGAACCG TAAAAAGGCC

2801 GCGTTGCTGG CGTTTTTCCA TAGGCTCCGC CCCCCTGACG AGCATCACAA AAATCGACGC TCAAGTCAGA GGTGGCGAAA

2881 CCCGACAGGA CTATAAAGAT ACCAGGCGTT TCCCCCTGGA AGCTCCCTCG TGCGCTCTCC TGTTCCGACC CTGCCGCTTA

2961 CCGGATACCT GTCCGCCTTT CTCCCTTCGG GAAGCGTGGC GCTTTCTCAT AGCTCACGCT GTAGGTATCT CAGTTCGGTG

3041 TAGGTCGTTC GCTCCAAGCT GGGCTGTGTG CACGAACCCC CCGTTCAGCC CGACCGCTGC GCCTTATCCG GTAACTATCG

3121 TCTTGAGTCC AACCCGGTAA GACACGACTT ATCGCCACTG GCAGCAGCCA CTGGTAACAG GATTAGCAGA GCGAGGTATG

3201 TAGGCGGTGC TACAGAGTTC TTGAAGTGGT GGCCTAACTA CGGCTACACT AGAAGAACAG TATTTGGTAT CTGCGCTCTG

3281 CTGAAGCCAG TTACCTTCGG AAAAAGAGTT GGTAGCTCTT GATCCGGCAA ACAAACCACC GCTGGTAGCG GTGGTTTTTT

3361 TGTTTGCAAG CAGCAGATTA CGCGCAGAAA AAAAGGATCT CAAGAAGATC CTTTGATCTT TTCTACGGGG TCTGACGCTC

3441 AGTGGAACGA AAACTCACGT TAAGGGATTT TGGTCATGAG ATTATCAAAA AGGATCTTCA CCTAGATCCT TTTAAATTAA

3521 AAATGAAGTT TTAAATCAAT CTAAAGTATA TATGAGTAAA CTTGGTCTGA CAGTTACCAA TGCTTAATCA GTGAGGCACC

3601 TATCTCAGCG ATCTGTCTAT TTCGTTCATC CATAGTTGCC TGACTCCCCG TCGTGTAGAT AACTACGATA CGGGAGGGCT

3681 TACCATCTGG CCCCAGTGCT GCAATGATAC CGCGAGACCC ACGCTCACCG GCTCCAGATT TATCAGCAAT AAACCAGCCA

3761 GCCGGAAGGG CCGAGCGCAG AAGTGGTCCT GCAACTTTAT CCGCCTCCAT CCAGTCTATT AATTGTTGCC GGGAAGCTAG

3841 AGTAAGTAGT TCGCCAGTTA ATAGTTTGCG CAACGTTGTT GCCATTGCTA CAGGCATCGT GGTGTCACGC TCGTCGTTTG

3921 GTATGGCTTC ATTCAGCTCC GGTTCCCAAC GATCAAGGCG AGTTACATGA TCCCCCATGT TGTGCAAAAA AGCGGTTAGC

4001 TCCTTCGGTC CTCCGATCGT TGTCAGAAGT AAGTTGGCCG CAGTGTTATC ACTCATGGTT ATGGCAGCAC TGCATAATTC

4081 TCTTACTGTC ATGCCATCCG TAAGATGCTT TTCTGTGACT GGTGAGTACT CAACCAAGTC ATTCTGAGAA TAGTGTATGC
4161 GGCGACCGAG TTGCTCTTGC CCGGCGTCAA TACGGGATAA TACCGCGCCA CATAGCAGAA CTTTAAAAGT GCTCATCATT
4241 GGAAAACGTT CTTCGGGGCG AAAACTCTCA AGGATCTTAC CGCTGTTGAG ATCCAGTTCG ATGTAACCCA CTCGTGCACC
4321 CAACTGATCT TCAGCATCTT TTACTTTCAC CAGCGTTTCT GGGTGAGCAA AAACAGGAAG GCAAAATGCC GCAAAAAAGG
4401 GAATAAGGGC GACACGGAAA TGTTGAATAC TCATACTCTT CCTTTTTCAA TATTATTGAA GCATTTATCA GGGTTATTGT
4481 CTCATGAGCG GATACATATT TGAATGTATT TAGAAAAATA AACAAATAGG GGTTCCGCGC ACATTTCCCC GAAAAGTGCC
4561 ACCTGACGTC TAAGAAACCA TTATTATCAT GACATTAACC TATAAAAATA GGCGTATCAC GAGGCCCTTT CGTCGGCGCG
4641 CCGCGGCCGC CAACTTTGTA TAGAAAAGTT GCTGTGCCTT CTAGTTGCCA GCCATCTGTT GTTTGCCCCT CCCCCGTGCC
4721 TTCCTTGACC CTGGAAGGTG CCACTCCCAC TGTCCTTTCC TAATAAAATG AGGAAATTGC ATCGCATTGT CTGAGTAGGT
4801 GTCATTCTAT TCTGGGGGGT GGGGTGGGGC AGGACAGCAA GGGGGAGGAT TGGGAAGACA ATAGCAGGCA TGCTGGGGAT
4881 GCGGTGGGCT CTATGG

Vector name – MyoG-mutVDRE-GFP

Name	Position	Size (bp)	Type	Description	Application notes
Myogenin promoter including mutated VDRE	1-1577	1577	Promoter	Promoter sequence for mouse gene Myogenin	Region of DNA upstream from the Myogenin coding sequence where relevant proteins bind to initiate or repress transcription of the gene.
Kozak	1608-1613	6	Miscellaneous	Kozak translation initiation sequence	Facilitates translation initiation of ATG start codon downstream of the Kozak sequence.
TurboGFP	1614-2297	684	ORF	Also known as maxGFP; green fluorescent protein from maxillopoda	Fast maturation; ranked high in brightness, photostability and pH stability among all fluorescent proteins.
SV40 late pA	2398-2619	222	PolyA_signal	Simian virus 40 late polyadenylation signal	Allows transcription termination and polyadenylation of mRNA transcribed by Pol II RNA polymerase.
pUC ori	complement (2815-3403)	589	Rep_origin	pUC origin of replication	Facilitates plasmid replication in E. coli; regulates high-copy plasmid number (500-700).
Ampicillin	complement (3574-4434)	861	ORF	Ampicillin resistance gene	Allows E. coli to be resistant to ampicillin.

Sequence:

```
  1 TCTAGAGTTG TATGACGCAG GCAAAGGTGA TGACTCAGGC AGGAAGGAAT AGAAGAGGCC AGCCTGGTGG CCCAGGACAG
 81 ACAAATGATG CAAAGGACTC TTTCTCCTTA TCGACCCTTC TACAGAAAGG AAAGAGTCAA AACGGTTCTA GTGCCAGAAG
161 GCATTATTGA GGGGAAAGCA CAGAAGAGAT GATTAAGAGC ATCAGACAGG GTCCATCCCA TAATCAGCCA CCAAACACAG
241 ACACTATTAC ATATGCCAGC AAGATTTTGC TGAAAGAACC CTGATATAGC TGCCTCTTGT GAGGCTATGC AGTGCCTGGC
321 AAATACAGAA GTGGATGCTC ACAGTCAGCT ATTGGATGAG GCACAGGGCC CCCAATGGAG GAGCTAGAGA AAGTACCCAA
401 GGAACTGAAG GGGTCTGCAA CCCTATAGGT GGAACAACAA TATGAACTAA CCAGTACCCC CAGAGCTCAT GTCTCTAGCT
481 GCATATGTAG CAGAAGATGG CCTAGTCGGC CATCATTGGG AAGAGAGGCC CCTTGGTATT GCAAACTATA TGCCCCAGTA
561 CAGGGGAACG CCAGGGCCAA GAAGTGGGAA TGAGTGGGTA GGGGAGCAGG GCGGGGGGAG GGGGGGGTAT AGGGAACTTT
641 TGGGATAGCA TTTGAAATGT AAATGAAGAA AATATCTAAT AAAAAATAAT TTAAAAAAGA GCGTCAGACA GGGGACTGAA
721 CAGCTCTTGC ACTAGGGGAG AAGAAGGCAA TGTAGAGTAG TCTGTGAGTT CTAATCCTTG CTAAACACTG ACTTCACCTG
801 ACCCCTACTA CTTAAGGCCC CCCCCCCTTA CTTAAGAAGT CCCTGTGTTC TCTTACTTCA ATCTACCCCC AACATCATGA
881 GACCTGGTCA AAGAAGCTGT AGAAACCCAA AAGTTGAATC CATTTGCCCT TCTGGGTTTC TGTCTTTGCC TCCATGGACG
```

```
961  ATAGGACACA CACACACACA CACACACACA CACACACACA CACACACACA CGCCCCAAAT CTGGAGTGGT CCTGATGTGG
1041 TAGTGGTAGG TCTTTAGGGG TCTCATGGGA CTGACATAGT ACGGTTTAAG GTGCTGCTGC TGAGCAGGAA AGAGAAGGCT
1121 AAGTGGATTT TCAAGACCCC TTCCCGTCCG TCCAAGACAA CCCCTTTCTT GTTCCCTTCC TGCCCTGTCC ACCAGCTGCC
1201 TTGGACCATG GAGGAGAGAG TAGGCAGGAG GCCCGGGTAG GAGTAATTGA AAGGAGCAGA TGAGACGGGG GAATGCACCC
1281 ACCCCCACCT TCCCTGCCCC ACAGGCTGTG GAGAAATGAA AACTAATCAA ATTACAGCCG ACGGCCTCCC GACCCGTGCA
1361 CAGGAGCCGC CTGGGCCAGG GGCAGGCCTG CAGGGTGGGG TGGGGGCAAA AGGAGAGGGA AGGGGAATCA CATGTAATCC
1441 ACTGGAAACG TCTTGATGTG CAGCAACAGC TTAGAGGGGG GCTCAGGTTT CTGTGGCGTT GGCTATATTT ATCTCTGGGT
1521 TCATGCCAGC AGGGAGGGTT TAAATGGCAC CCAGCAGTTG GCGTGAGGGG CTGCGGGTCT AGACAAGTTT GTACAAAAAA
1601 GCAGGCTGCC ACCATGCCCG CCATGAAGAT CGAGTGCCGC ATCACCGGCA CCCTGAACGG CGTGGAGTTC GAGCTGGTGG
1681 GCGGCGGAGA GGGCACCCCC GAGCAGGGCC GCATGACCAA CAAGATGAAG AGCACCAAAG GCGCCCTGAC CTTCAGCCCC
1761 TACCTGCTGA GCCACGTGAT GGGCTACGGC TTCTACCACT TCGGCACCTA CCCCAGCGGC TACGAGAACC CCTTCCTGCA
1841 CGCCATCAAC AACGGCGGCT ACACCAACAC CCGCATCGAG AAGTACGAGG ACGGCGGCGT GCTGCACGTG AGCTTCAGCT
1921 ACCGCTACGA GGCCGGCCGC GTGATCGGCG ACTTCAAGGT GGTGGGCACC GGCTTCCCCG AGGACAGCGT GATCTTCACC
2001 GACAAGATCA TCCGCAGCAA CGCCACCGTG GAGCACCTGC ACCCCATGGG CGATAACGTG CTGGTGGGCA GCTTCGCCCG
2081 CACCTTCAGC CTGCGCGACG GCGGCTACTA CAGCTTCGTG GTGGACAGCC ACATGCACTT CAAGAGCGCC ATCCACCCCA
2161 GCATCCTGCA GAACGGGGGC CCCATGTTCG CCTTCCGCCG CGTGGAGGAG CTGCACAGCA ACACCGAGCT GGGCATCGTG
2241 GAGTACCAGC ACGCCTTCAA GACCCCCATC GCCTTCGCCA GATCTCGAGC TCGATGACGC ACCAAGGAAG CCCTCGAGGA
2321 CGCGTAAAGG TACCAAAGGA TCCCGACCTA CCGACCCAGC TTTCTTGTAC AAAGTGGTGA TGGCCGGCCG CTTCGAGCAG
2401 ACATGATAAG ATACATTGAT GAGTTTGGAC AAACCACAAC TAGAATGCAG TGAAAAAAAT GCTTTATTTG TGAAATTTGT
2481 GATGCTATTG CTTTATTTGT AACCATTATA AGCTGCAATA AACAAGTTAA CAACAACAAT TGCATTCATT TTATGTTTCA
2561 GGTTCAGGGG GAGGTGTGGG AGGTTTTTTA AAGCAAGTAA AACCTCTACA AATGTGGTAG CGGCCGCGGC GCTCTTCCGC
2641 TTCCTCGCTC ACTGACTCGC TGCGCTCGGT CGTTCGGCTG CGGCGAGCGG TATCAGCTCA CTCAAAGGCG GTAATACGGT
2721 TATCCACAGA ATCAGGGGAT AACGCAGGAA AGAACATGTG AGCAAAAGGC CAGCAAAAGG CCAGGAACCG TAAAAAGGCC
2801 GCGTTGCTGG CGTTTTTCCA TAGGCTCCGC CCCCCTGACG AGCATCACAA AAATCGACGC TCAAGTCAGA GGTGGCGAAA
2881 CCCGACAGGA CTATAAAGAT ACCAGGCGTT TCCCCCTGGA AGCTCCCTCG TGCGCTCTCC TGTTCCGACC CTGCCGCTTA
2961 CCGGATACCT GTCCGCCTTT CTCCCTTCGG GAAGCGTGGC GCTTTCTCAT AGCTCACGCT GTAGGTATCT CAGTTCGGTG
3041 TAGGTCGTTC GCTCCAAGCT GGGCTGTGTG CACGAACCCC CCGTTCAGCC CGACCGCTGC GCCTTATCCG GTAACTATCG
3121 TCTTGAGTCC AACCCGGTAA GACACGACTT ATCGCCACTG GCAGCAGCCA CTGGTAACAG GATTAGCAGA GCGAGGTATG
3201 TAGGCGGTGC TACAGAGTTC TTGAAGTGGT GGCCTAACTA CGGCTACACT AGAAGAACAG TATTTGGTAT CTGCGCTCTG
3281 CTGAAGCCAG TTACCTTCGG AAAAAGAGTT GGTAGCTCTT GATCCGGCAA ACAAACCACC GCTGGTAGCG GTGGTTTTTT
3361 TGTTTGCAAG CAGCAGATTA CGCGCAGAAA AAAAGGATCT CAAGAAGATC CTTTGATCTT TTCTACGGGG TCTGACGCTC
3441 AGTGGAACGA AAACTCACGT TAAGGGATTT TGGTCATGAG ATTATCAAAA AGGATCTTCA CCTAGATCCT TTTAAATTAA
3521 AAATGAAGTT TTAAATCAAT CTAAAGTATA TATGAGTAAA CTTGGTCTGA CAGTTACCAA TGCTTAATCA GTGAGGCACC
3601 TATCTCAGCG ATCTGTCTAT TTCGTTCATC CATAGTTGCC TGACTCCCCG TCGTGTAGAT AACTACGATA CGGGAGGGCT
3681 TACCATCTGG CCCCAGTGCT GCAATGATAC CGCGAGACCC ACGCTCACCG GCTCCAGATT TATCAGCAAT AAACCAGCCA
3761 GCCGGAAGGG CCGAGCGCAG AAGTGGTCCT GCAACTTTAT CCGCCTCCAT CCAGTCTATT AATTGTTGCC GGGAAGCTAG
3841 AGTAAGTAGT TCGCCAGTTA ATAGTTTGCG CAACGTTGTT GCCATTGCTA CAGGCATCGT GGTGTCACGC TCGTCGTTTG
3921 GTATGGCTTC ATTCAGCTCC GGTTCCCAAC GATCAAGGCG AGTTACATGA TCCCCCATGT TGTGCAAAAA AGCGGTTAGC
4001 TCCTTCGGTC CTCCGATCGT TGTCAGAAGT AAGTTGGCCG CAGTGTTATC ACTCATGGTT ATGGCAGCAC TGCATAATTC
4081 TCTTACTGTC ATGCCATCCG TAAGATGCTT TTCTGTGACT GGTGAGTACT CAACCAAGTC ATTCTGAGAA TAGTGTATGC
4161 GGCGACCGAG TTGCTCTTGC CCGGCGTCAA TACGGGATAA TACCGCGCCA CATAGCAGAA CTTTAAAAGT GCTCATCATT
4241 GGAAAACGTT CTTCGGGGCG AAAACTCTCA AGGATCTTAC CGCTGTTGAG ATCCAGTTCG ATGTAACCCA CTCGTGCACC
4321 CAACTGATCT TCAGCATCTT TTACTTTCAC CAGCGTTTCT GGGTGAGCAA AAACAGGAAG GCAAAATGCC GCAAAAAAGG
4401 GAATAAGGGC GACACGGAAA TGTTGAATAC TCATACTCTT CCTTTTTCAA TATTATTGAA GCATTTATCA GGGTTATTGT
4481 CTCATGAGCG GATACATATT TGAATGTATT TAGAAAAATA AACAAATAGG GGTTCCGCGC ACATTTCCCC GAAAAGTGCC
4561 ACCTGACGTC TAAGAAACCA TTATTATCAT GACATTAACC TATAAAAATA GGCGTATCAC GAGGCCCTTT CGTCGGCGCG
```

4641 CCGCGGCCGC CAACTTTGTA TAGAAAAGTT GCTGTGCCTT CTAGTTGCCA GCCATCTGTT GTTTGCCCCT CCCCCGTGCC
4721 TTCCTTGACC CTGGAAGGTG CCACTCCCAC TGTCCTTTCC TAATAAAATG AGGAAATTGC ATCGCATTGT CTGAGTAGGT
4801 GTCATTCTAT TCTGGGGGGT GGGGTGGGGC AGGACAGCAA GGGGGAGGAT TGGGAAGACA ATAGCAGGCA TGCTGGGGAT
4881 GCGGTGGGCT CTATGG

Supplementary data 3 – Correlation between myogenin and MHC early isoforms

Supplementary data 3 shows correlation graphs for myogenin mRNA levels plotted against MHC I, MHC embryonic and MHC neonatal mRNA levels on day 6 and day 8 including the R^2 values which can be seen on the graphs. This includes all vitamin D treatment groups and controls.

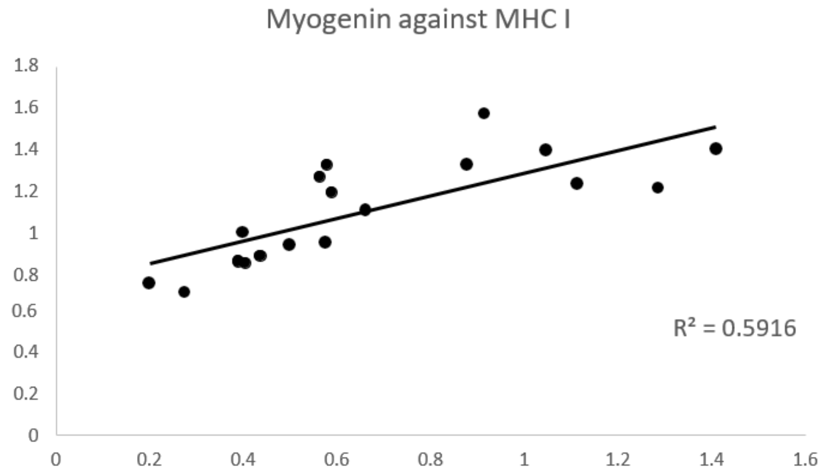

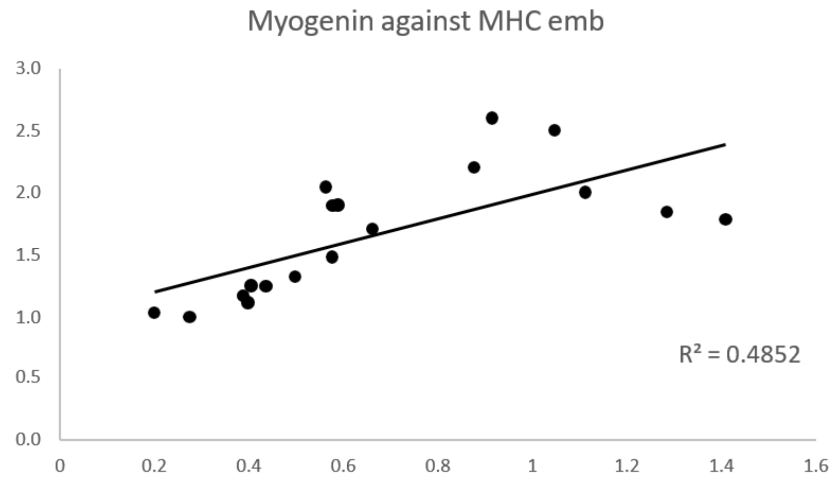

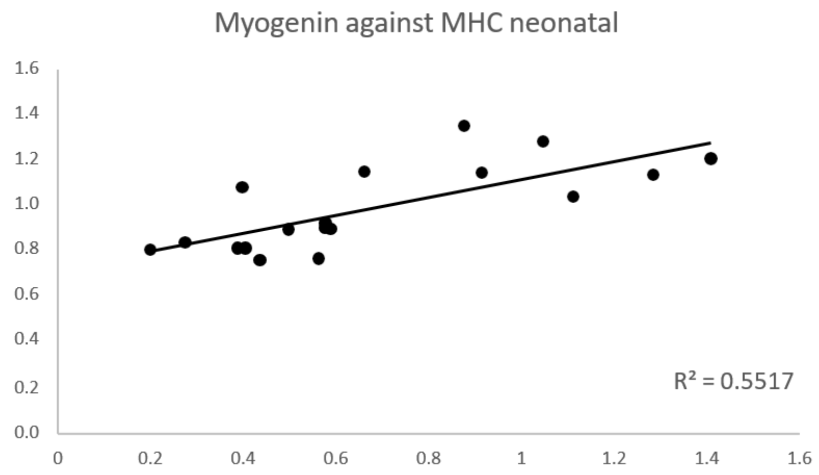

Supplementary data 4 – Cell Images

Supplementary data 4 shows cell images (at 10X magnification) of transfected C2C12 cells on days 1, 2, 5 and 8 (representing the beginning, early, mid and late stages of differentiation) taken with a fluorescent microscope. GFP (green cells) are transfected with the MyoG-VDRE-GFP vector which can be seen in supplementary data 2. Blue cells are stained with DAPI to give an indication of cell number. All GFP fluorescence results within this study were normalised to DAPI. RFP (red) cells were transfected with a vector containing a constitutively active CMV promoter followed by RFP which becomes expressed within a few hours of the vector being within the cells. This was to check for transfection efficiency after 24 hours. Due to myogenin not being expressed until later in differentiation, cells did not appear green within 24 hours post transfection, therefore the RFP vector was used to confirm that cells had transfected 24 hours post transfection.

Day 1

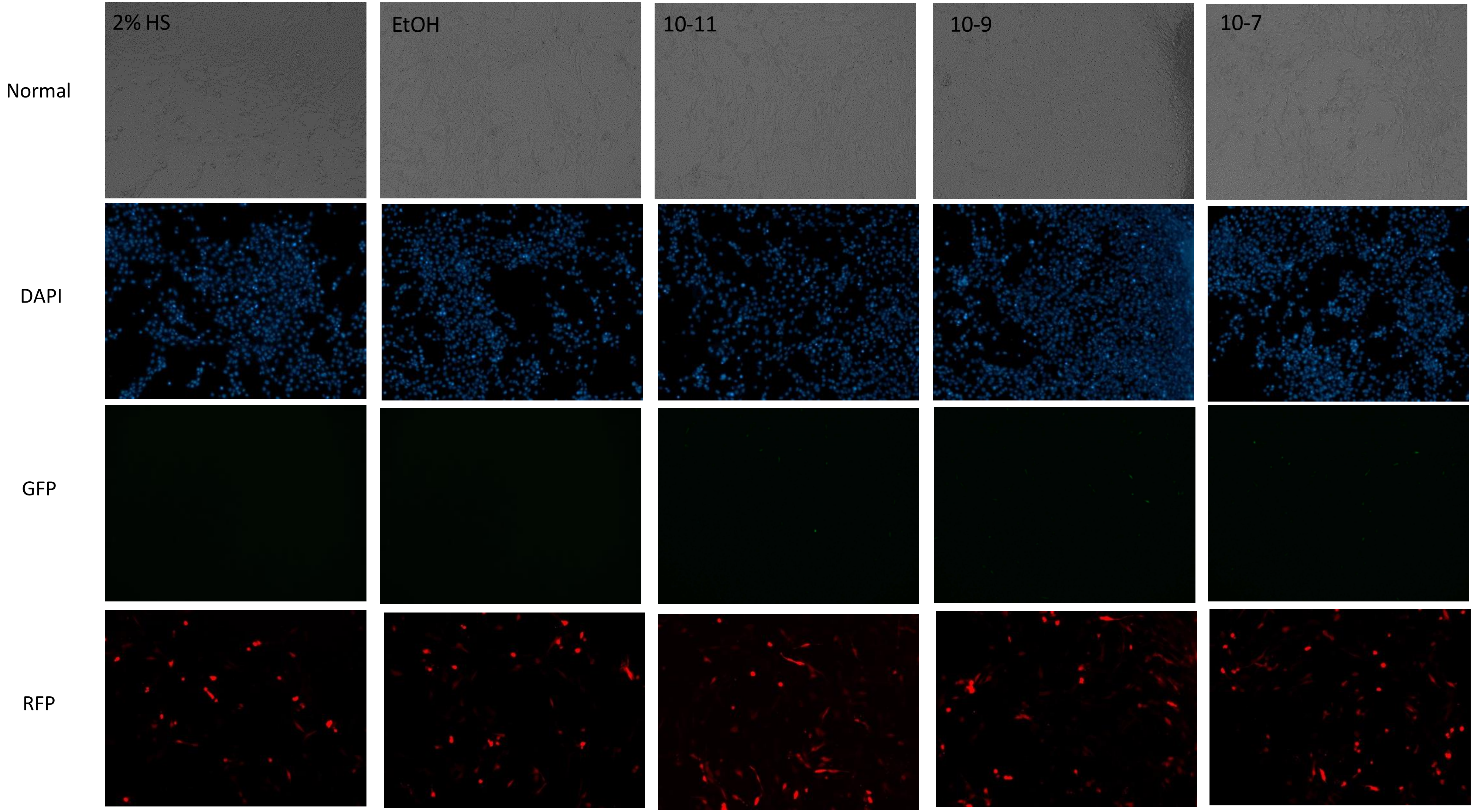

Day 2

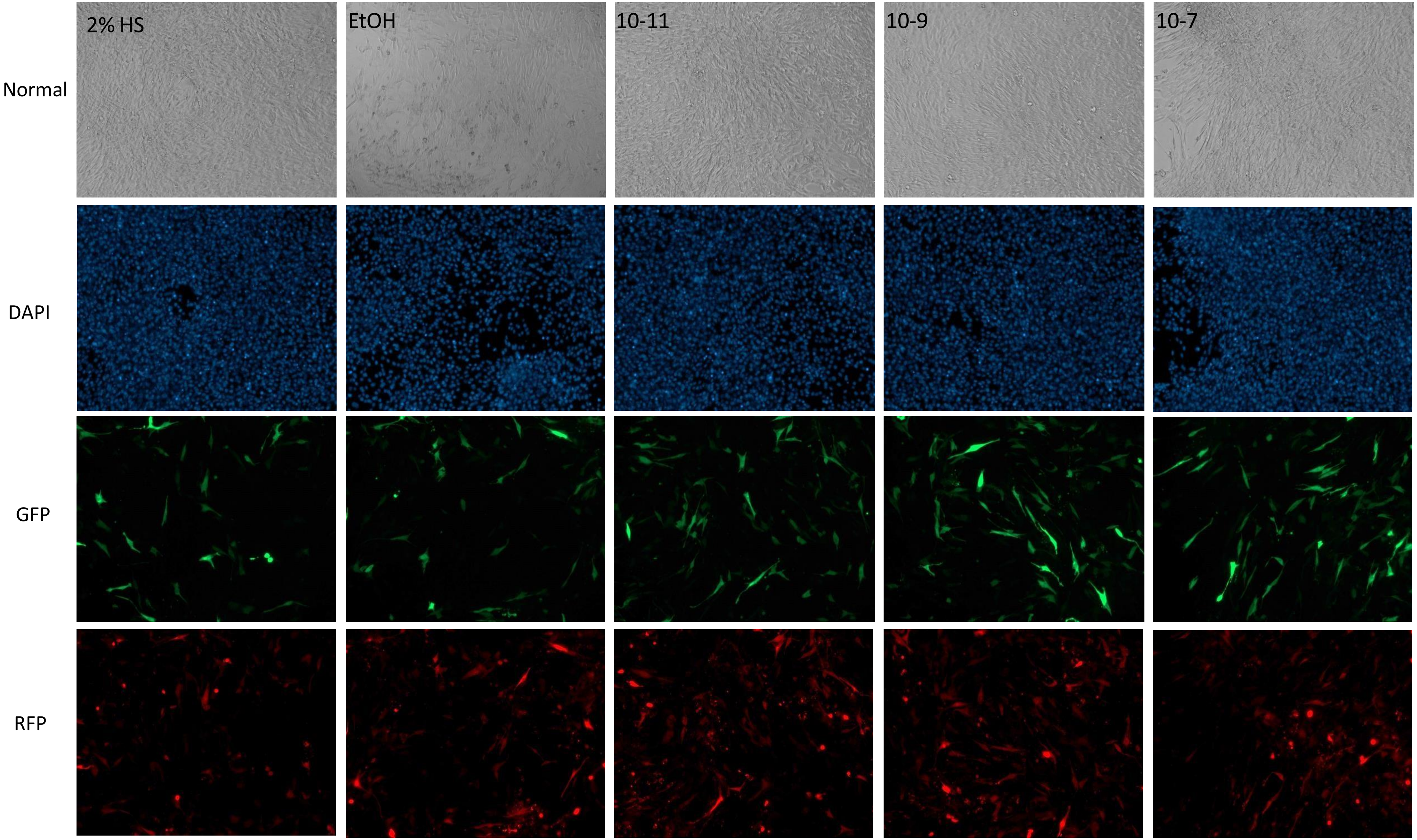

Day 5

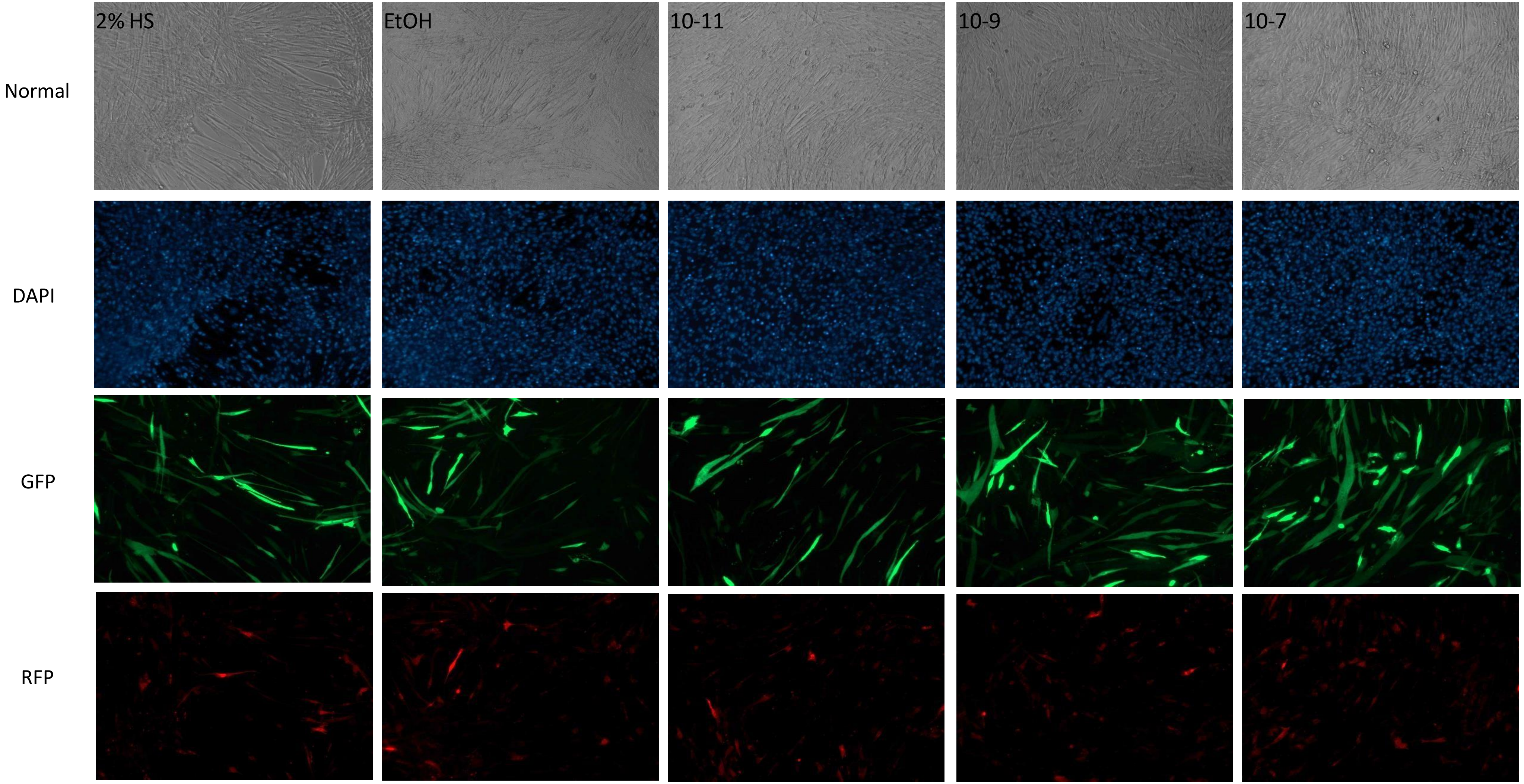

Day 8

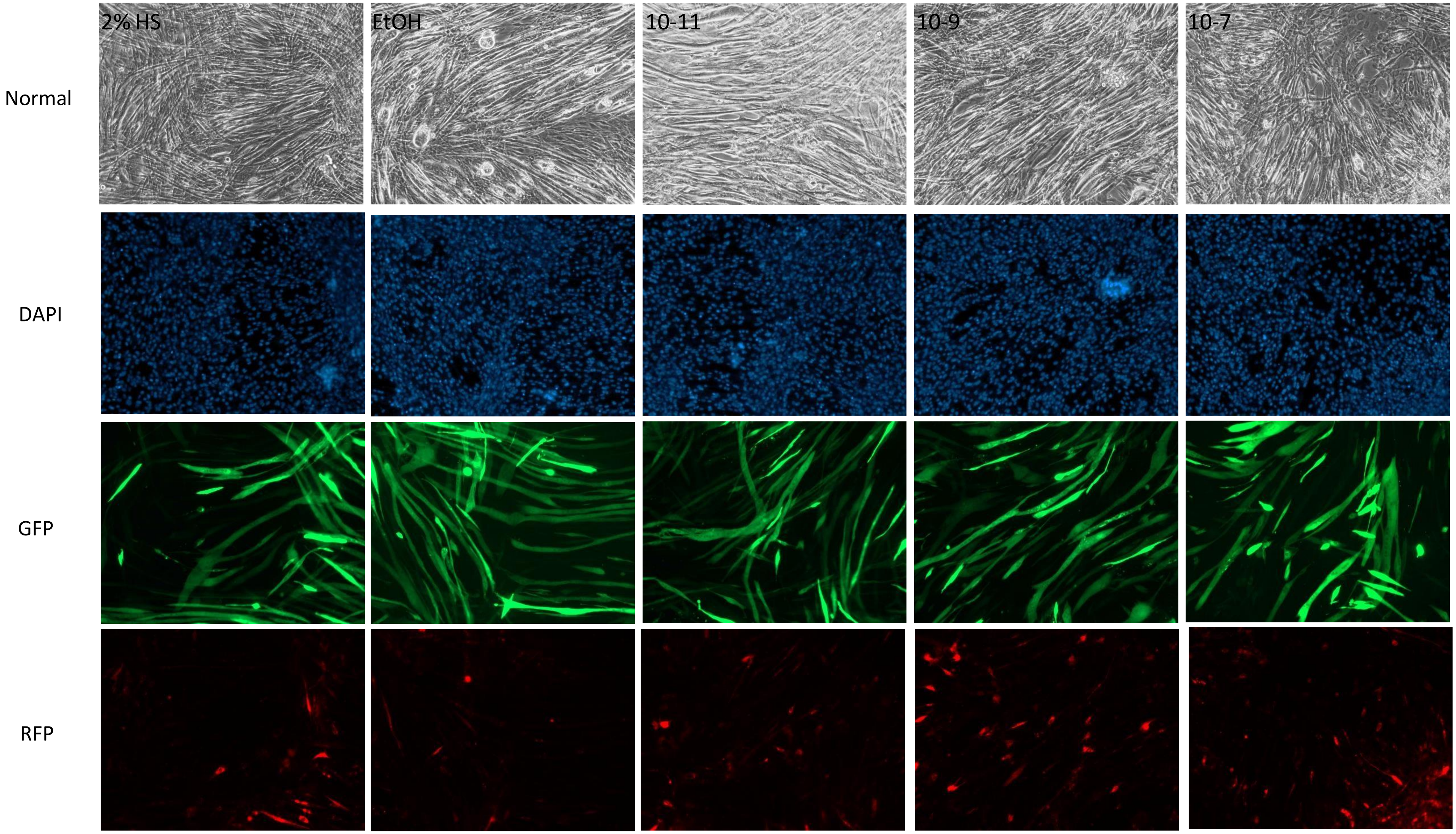

Supplementary data 5 – PCR products from ChIP

Supplementary data 5 shows a gel image of PCR products from the ChIP experiment run on a 2.5% metaphor agarose gel alongside a 25bp and 50bp ladder. Positive control was an RNA pol II antibody with GAPDH primers (provided in Chip kit) and negative control was a normal rabbit IgG antibody run with the VDRE primers (primer sequences can be viewed in supplementary data 1).

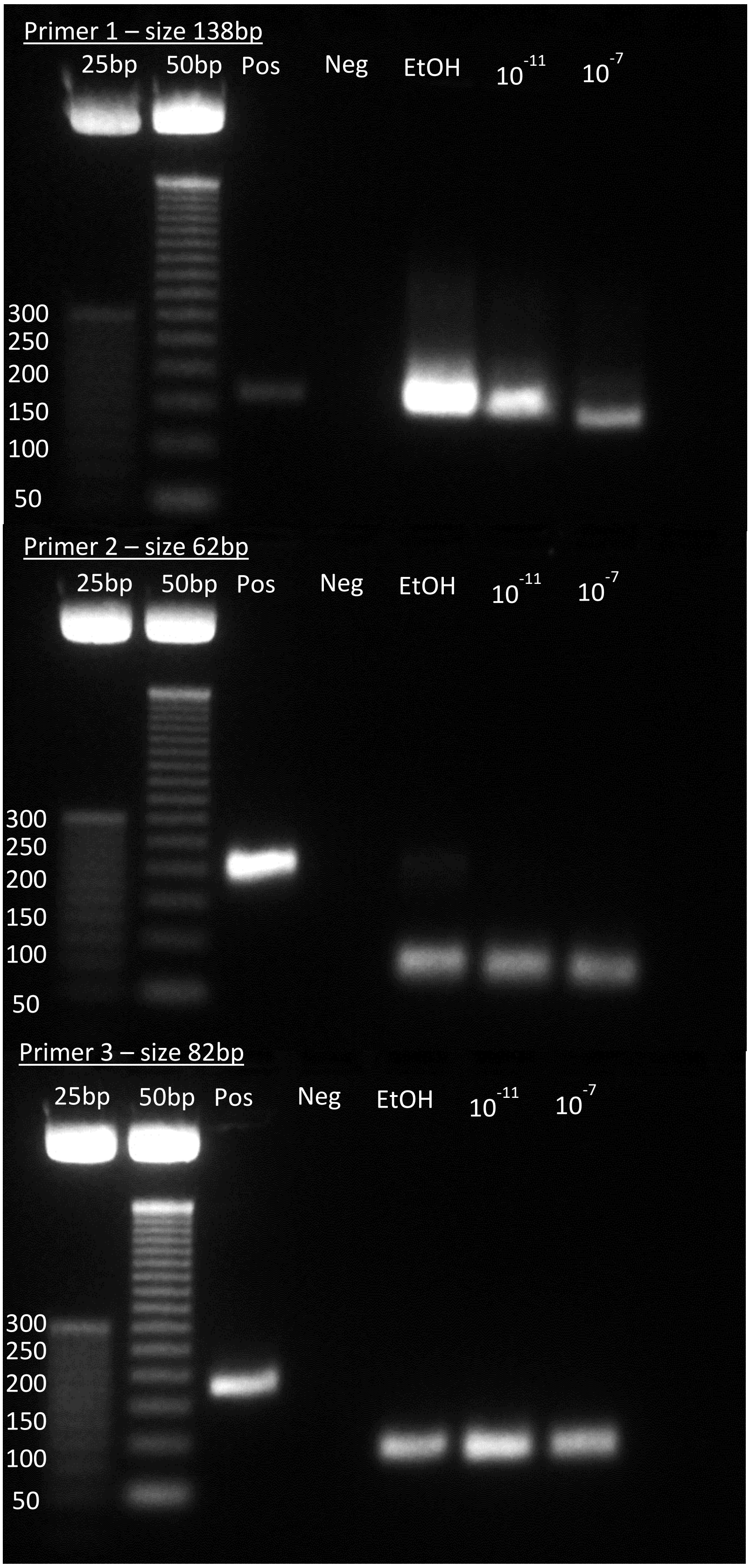

Supplement: Supplementary file 1 [file DataSheet1.PDF]
